# Influence of heterochirality on the structure, dynamics, biological properties of cyclic(PFPF) tetrapeptides obtained by solvent-free ball mill mechanosynthesis

**DOI:** 10.1038/s41598-024-63552-4

**Published:** 2024-06-04

**Authors:** Irena Bak-Sypien, Tomasz Pawlak, Piotr Paluch, Aneta Wroblewska, Rafał Dolot, Aleksandra Pawlowicz, Małgorzata Szczesio, Ewelina Wielgus, Sławomir Kaźmierski, Marcin Górecki, Roza Pawlowska, Arkadiusz Chworos, Marek J. Potrzebowski

**Affiliations:** 1 Centre of Molecular and Macromolecular Studies, Polish Academy of Sciences, Sienkiewicza 112 St., 90-363 Lodz, Poland; 2 Institute of Bioorganic Chemistry, Polish Academy of Sciences, Noskowskiego 12/14 St., 61-704 Poznan, Poland; 3 Institute of General and Ecological Chemistry, Faculty of Chemistry, Lodz University of Technology, Żeromskiego 116 St., 90-924 Lodz, Poland; 4 Institute of Organic Chemistry, Polish Academy of Sciences, Kasprzaka 44/52 St., 01-224 Warsaw, Poland

**Keywords:** Biochemistry, Chemical biology, Drug discovery

## Abstract

Cyclic tetrapeptides c(Pro-Phe-Pro-Phe) obtained by the mechanosynthetic method using a ball mill were isolated in a pure stereochemical form as a homochiral system (all *L*-amino acids, sample A) and as a heterochiral system with *D* configuration at one of the stereogenic centers of Phe (sample B). The structure and stereochemistry of both samples were determined by X-ray diffraction studies of single crystals. In DMSO and acetonitrile, sample A exists as an equimolar mixture of two conformers, while only one is monitored for sample B. The conformational space and energetic preferences for possible conformers were calculated using DFT methods. The distinctly different conformational flexibility of the two samples was experimentally proven by Variable Temperature (VT) and 2D EXSY NMR measurements. Both samples were docked to histone deacetylase HDAC8. Cytotoxic studies proved that none of the tested cyclic peptide is toxic.

## Introduction

Cyclic peptides, a class of compounds with a closed ring structure, have gained a lot of attention in many industrial and research fields due to their unique properties^1–8^. Their cyclic nature provides them with increased stability, target specificity, and often enhanced bioactivity compared to linear peptides^9–12^. Cyclic peptides are known to exhibit a wide spectrum of biological activities, such as antibacterial, antiviral, antifungal, anticancer and immunosuppressive^1,6,13–24^. Moreover, these compounds are characterized by additional resistance to enzymatic degradation by exoproteases that preferentially cleave near the N- or C-termini of peptides^25^. In the light of given *supra* information there is no surprise that over 60% of peptide drugs approved by FDA and EMA exist in the cyclic form^12,26^.

In recent review articles some exceptional properties of cyclic peptides have been discussed in detail^27–32^. Heinis and colleagues described therapeutically relevant cyclic peptides derived from natural sources, huge technological advances in the development of peptide ligands, and current challenges and opportunities for the development of cyclic peptides that address unmet medical needs^27^. An intriguing feature of cyclic peptides is their specific conformational flexibility. In this sense, this class of compounds belongs to the group of molecular chameleons. Kihlberg and co-workers have shown the importance of molecular chameleons in drug discovery and their role in protein–protein interactions^28^. This aspect was exhaustively discussed by Cheng et al.^29^ Protein–protein interactions (PPIs) control many basic biological pathways that are often misregulated in diseases^30–32^.

From a chemical point of view, cyclic peptides belong to the family of versatile complex structures. This complexity is due to the size of the structure determined by the number of amino acids in the sequence and the nature of the amino acids that make up the cyclic system. Among the 21 naturally occurring amino acids, proline plays a special role due to the unique structure associated with the existence of a five-membered ring in its side chain^33^. The presence of a five-membered ring limits the rotation around the nitrogen-carbon bond, which consequently has a significant impact on the secondary structure of peptides^34^. The conformational limitations of the peptide skeleton affect the folding into a typical alpha sheet of the helix or beta sheet^35^. Proline-rich regions in proteins are known to play an important role in protein–protein interactions, and proline is often found in turn-based alpha-helix regions and beta sheets. Therefore, proline is often used as an inducer forcing the peptide/protein to adopt the required higher-order structure^36^.

Proline-rich peptides (PRPs) are promising candidates for exploring the area of medical applications^37^. In particular these peptides in cyclic form (cPRPs) has received great deal of attention as potential, new generation drugs. It is worth noting that most cPRP has been isolated from marine organisms. The state of the art in this field has been reviewed by Zhang et al.^38^, as well as Fang et al.^39^ The biological activity of cPRPs were tested in different applications like: cytotoxicity, antibacterial, antifungal, immunosuppressive, anti-inflammatory, anti-HIV, repellent (antifouling), antitubercular and antiviral. It has been shown that cyclization can form peptides with the ability to penetrate tumors to increase the potency of anticancer drugs. Cyclic proline peptides can adopt desirable constrained geometries that are responsible for increasing their binding affinity, specificity or stability compared with their linear counterparts and might be more cell permeable due to the reduced conformational flexibility.

Proline and proline-rich cyclic peptides can occur in combination with other amino acids^40^. For example, the c(Pro-Phe-Asp) has been reported to exhibit anti-inflammatory activity by inhibiting the production of pro-inflammatory cytokines in macrophages. Cyclic dipeptide (Pro-Phe) has been reported to exhibit selective cytotoxicity against cancer cells, including breast cancer, colon cancer, and leukemia cells. The mechanism of action is thought to involve binding to the cell membrane and inducing cell death by disrupting the membrane integrity and causing the release of intracellular contents. Cyclic tripeptide c(Phe-Pro-Gly) has been found to exhibit cytotoxicity against colon cancer cells, while the cyclic peptide c(Pro-Phe-Gly) has been reported to exhibit cytotoxicity against leukemia cells. These results clearly prove that even very subtle structural changes can cause the significant differences in biological activity^41^.

In recent years much attention was paid to study of chemical modifications of cPRPs. Zabrocki and coworkers have patented method of synthesis of tetra-peptide mimetic Pro-Pro-Phe-β^3^hoPhe^42^. The advanced biological study proved that this compound is of a low toxicity and effective inhibitor of inflammatory disorders with potential therapeutic use, affecting the metabolism of prostanoid family molecules. Other analogues of c(Pro-Pro-Phe-Phe) were published by Bojarska et al.^43^.

In this work we present advanced structural and biological studies of cyclic tetrapeptide c(Pro-Phe-Pro-Phe). This tetrapeptide in homochiral form was originally isolated by Aracil et al. from the marine *ascidian Cystodytes dellechiajei*^44^. Cyclic tetrapeptide c(Pro-Phe-Pro-Phe) was also prepared in the chemical laboratory by surface cyclization of a linear tetrapeptide using molecularly printed polymers^45^. In both articles, the product was analyzed using standard approaches. In our article we show few hidden features of this system; structural complexity in function of chirality. For preparation of models we employed new approach, based on our original idea which employs synergy of mechanochemistry and crystal engineering.

## Results and discussion

### Synthesis and single crystal X-ray diffraction studies of cyclic PFPF tetrapeptides

One of the challenges of modern bioorganic chemistry is the effective synthesis of small cyclic peptides (SCP) containing from four to twelve amino acids in sequence^13,46^. Unfortunately, SCPs are difficult to synthesize. One of the reasons for the above-mentioned difficulties are cyclic constraints^47^. In general, because of energy constraints, medium-sized rings (9–12-membered rings) are more challenging to form compared to smaller (4–8-membered) and larger sized rings (> 12 members). This is mainly due to the fact that the additional transannular interactions between substituents of these medium sized rings (between substituents of nonadjacent carbon atoms) play an important role in the ring strain energy. Different synthetic strategies for producing SCP were recently reviewed by Sarojini et al.^48^. For the successful synthesis of any cyclic peptide in the solution, the N- and C-termini of the linear peptide chain must be in close spatial proximity for intramolecular cyclization. For solution phase cyclizations several factors play an important role in achieving success with cyclomonomer formation against the competing intermolecular reactions leading to dimer, oligomer, or polymer formation. In particular, the choice of the coupling reagents, order of addition of reagents, and knowledge about their interrelationship are crucial. The reagents can be added to a solution of the peptide placed under high dilution or to a solution of the reagents under stirring, the peptide solution can be added incrementally from a syringe pump. Solid-state mechanochemical synthesis can be considered an attractive alternative to the known “wet” procedures for economic and ecological reasons^49–51^.

The cyclic tetrapeptides c(Pro-Phe-Pro-Phe) were obtained using the mechanosynthetic approach described in our European Patent (EP number 4253398)^52^. This method of synthesis uses a specific pseudocyclic arrangement of linear tetrapeptides in a crystallographic unit cell, which consequently results in relatively short intramolecular and/or intermolecular distances between the active C and N ends involved in the formation of new peptide bonds. Such an example of a compound with pseudocyclic conformation in a crystal lattice, which was used in our project, is hydrochloride of Pro-Phe-Pro-Phe linear tetrapeptide. Its X-ray structure is deposited at the Cambridge Crystallographic Data Center (CCDC 2143095). Mechanochemical cyclization was carried out in vibratory mixer mill. The final products of pre-purified reaction mixture were separated employing flash chromatography (see Supplementary Information, Figs. S1 and S2). Two samples of cyclic cPFPF tetrapeptides were isolated (see Supplementary Information, Figs. S3 and S4). Both samples are soluble in organic solvents (acetonitrile, DMSO, chloroform, methanol) however are not soluble in water. As we found by testing various solvents, methanol was the optimal medium for growing c(Pro-Phe-Pro-Phe) single crystals. The crystallographic details and the refinement parameters for the crystalline forms of c(Pro-Phe-Pro-Phe) (sample **A**) and c(Pro-Phe-Pro-*D)*-Phe) (sample **B**) are given in Table 1, while all other data can be found in the “Supplementary Information”.

**Table 1:** Crystal structure, data collection and refinement parameters for c(Pro-Phe-Pro-Phe) (sample **A**) and c(Pro-Phe-Pro-*D*-Phe) (sample **B**), respectively.

Compound	c(Pro-Phe-Pro-Phe)	c(Pro-Phe-Pro-*D*-Phe)
Crystal data		
CCDC	2,330,472	2,330,474
Chemical formula	C_28_H_32_N_4_O_4_	C_28_H_32_N_4_O_4_
Formula weight	488.57	488.57
Crystal system	Monoclinic	Monoclinic
Space group	*P*2_1_	*P*2_1_
Temperature (K)	99.99(11)	100.00(10)
*a* [Å]	12.9381(1)	12.8531(2)
*b* [Å]	5.2726(1)	5.2299(1)
*c* [Å]	17.5667(2)	18.4907(3)
*β* [°]	101.721(1)	107.753(2)
*V* [Å^3^]	1173.37(3)	1183.76(4)
Z	2	2
Z’	1	1
d_calc_ [g/cm^3^]	1.383	1.371
Crystal dimensions [mm]	0.7 × 0.15 × 0.04	0.7 × 0.1 × 0.02
Radiation type	CuKα	CuKα
*μ* [mm^−1^]	0.758	0.751
Data collection		
Reflections measured	32,081	31,745
Range/indices (*h*, *k*, *l*)	− 16, 16	
− 6, 4		
− 21, 21	− 16, 16	
− 6, 4		
− 22, 22		
θ (max, min) [°]	75.881, 2.569	75.632, 2.509
Total no. of unique data	4244	4259
No. of observed data, I > 2σ(I)	4071	4078
*R*_int_	0.049	0.051
Refinement		
*R* [*F*^2^ > 2σ (*F*^2^)]	0.036	0.041
*wR*(*F*^2^)	0.098	0.098
*S*	1.096	1.040
No. of reflections	4244	4259
No. of parameters	333	334
No. of restraints	1	1
H-atom treatment	H atoms treated by a mixture of independent and constrained refinement	H atoms treated by a mixture of independent and constrained refinement
Δρ (min, max), e/Å^3^	− 0.24, 0.19	− 0.20, 0.38
Absolute structure parameter	− 0.07(11)	0.22(13)

**Figure 1:**
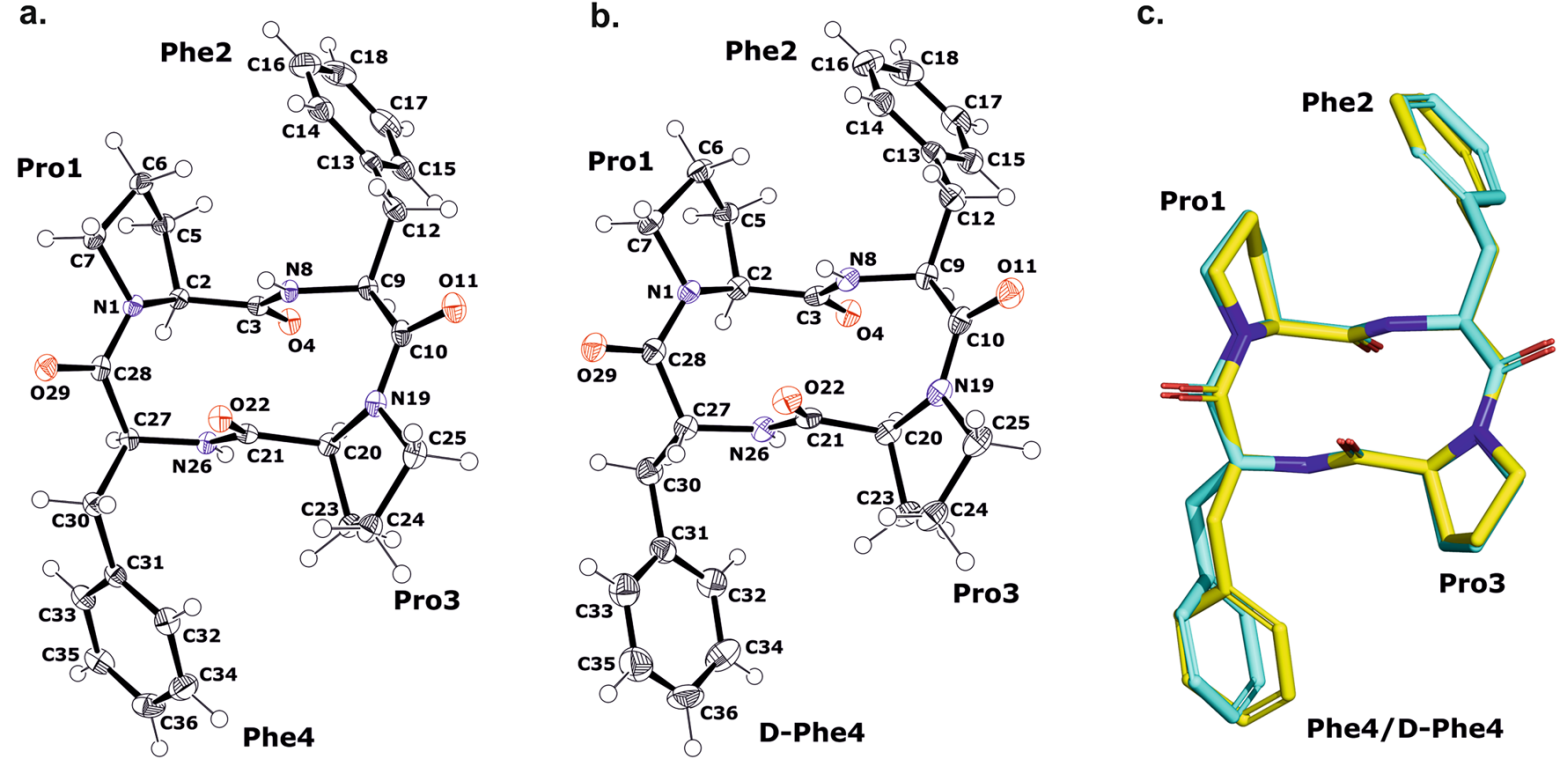
(**a**,**b**) The molecular structures of c(Pro-Phe-Pro-Phe) (sample A) and c(Pro-Phe-Pro-*D*-Phe) (sample B) crystals showing the atom-labeling scheme. The displacement ellipsoids are drawn with 50% probability level and the H atoms are shown as small spheres of arbitrary radius. (**c**) The superposition of the analyzed cyclic peptide molecules; c(Pro-Phe-Pro-Phe) and c(Pro-Phe-Pro-*D*-Phe) are shown as yellow and cyan, respectively.

X-ray crystallographic analysis revealed that the c(Pro-Phe-Pro-Phe) and c(Pro-Phe-Pro-*D*-Phe) compounds crystallize in the monoclinic space group *P*2_1_ and their asymmetric units consist of a single molecule of the cyclic peptide (see Supplementary Information, Fig. S5a and S5b). The geometry parameters of both molecules are almost identical, with the exception of the phenylalanyl residue at position 4, which adopts different conformations (Fig. 1c, see also Supplementary Information, Table S1–S6,). No intramolecular hydrogen bonds are observed within single cyclic peptide molecules, but they are formed with symmetrically related molecules. The geometrical data of the H-bonds are summarized in Table 2. The architecture of the crystal network is characterized by intermolecular H-bonds between amine and carbonyl groups (NH···O) of the neighboring symmetrically related molecules and by hydrophobic interactions formed by phenylalanine residues. The main NH···O bonds connect the molecules along the crystallographic b-axis and form a 1D structure. The C=O and NH groups of the peptides are localized on opposite sides of the backbone, resulting in a columnar stacking of molecules connected by intermolecular NH···O interactions, forming a kind of nanotube with an inner diameter of 5.5 Å × 2.3 Å.

**Table 2:** Hydrogen bond information for c(Pro-Phe-Pro-Phe) and c(Pro-Phe-Pro-*D*-Phe), respectively.

D-H	A	d(D-H) [Å]	d(H-A) [Å]	d(D-A) [Å]	D-H-A [°]
c(Pro-Phe-Pro-Phe)					
N8-H8^i^	O4	0.86(3)	2.30(3)	3.009(3)	28.9(19)
N26-H26	O22^i^	0.85(3)	2.25(3)	3.056(3)	15.0(17)
c(Pro-Phe-Pro-*D*-Phe)					
N8-H8^i^	O4	0.84(4)	2.26(4)	2.968(3)	142(3)
N26-H26	O22^i^	0.81(4)	2.30(4)	3.100(4)	5(2)

Symmetry codes: (i) + x, -1 + y, + z.

### 1D and 2D liquid state NMR studies

**Figure 2:**
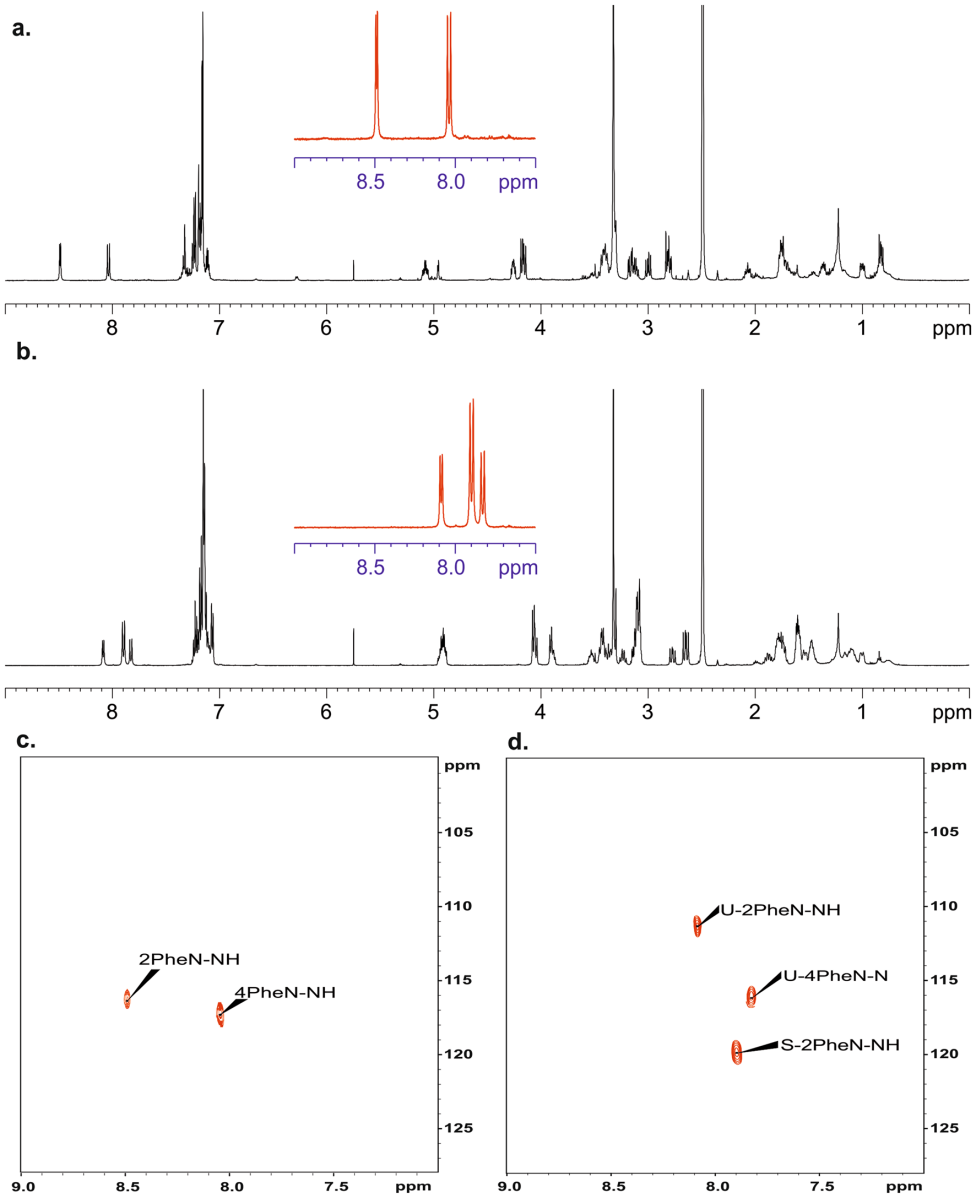
500 MHz ^1^H NMR spectra of sample B (**a**) and sample A (**b**) dissolved in DMSO-d_6_. The red inset shown in the middle of the spectrum represents N–H protons. The spectra were recorded at a temperature of 298 K. ^1^H–^15^N HSQC 2D NMR correlations for sample B (**c**) and for sample A (**d**). Samples were dissolved in DMSO-d_6_. Symbol S (**d**) represents sample with symmetrical geometry, symbol U represents unsymmetrical sample.

Having in mind the similarity in X-ray structure of sample **A** and **B** we expected also similarity in NMR spectral patterns. Figure 2a,b shows the ^1^H NMR spectra recorded in DMSO-d6. At first glance and from a brief comparison of the spectra, one can see that they are significantly different. The most spectacular differences are seen in region of N–H protons (9.0–7.5 ppm). In both spectra, this region is shown as a red inset in the central part. The spectral pattern for the **B** sample with stereochemistry *D* at one of the chiral centres of Phe is not surprising. As predicted, two doublets at δ = 8.49 ppm and δ = 8.04 ppm with vicinal ^3^J_H–H_ coupling constants equal to 4.79 Hz and 10.62 Hz can be assigned to the structure. The interpretation for sample **A** (all chiral centres are *L*) is more troublesome. In this case, in the N–H region, we see three doublets with chemical shifts at δ = 8.09 ppm, δ = 7.90 ppm, and δ = 7.81 ppm. The vicinal ^3^J_H–H_ coupling constants are found to be 7.31 Hz, 9.67 Hz and 9.93 Hz, respectively. The intensity of the middle signal is twice as high as the other NH signals.

Differences are also seen in the chemical shifts of ^15^N nucleus. Figure 2c,d shows the 2D^1^H–^15^N NMR heteronuclear correlations. For sample **B**, the chemical shifts are equal δ = 115.7 ppm and δ = 116.5 ppm while for sample **A** three signals are seen with ^15^N chemical shifts δ = 110.9 ppm, δ = 119.8 ppm and δ = 115.8 ppm.

**Figure 3:**
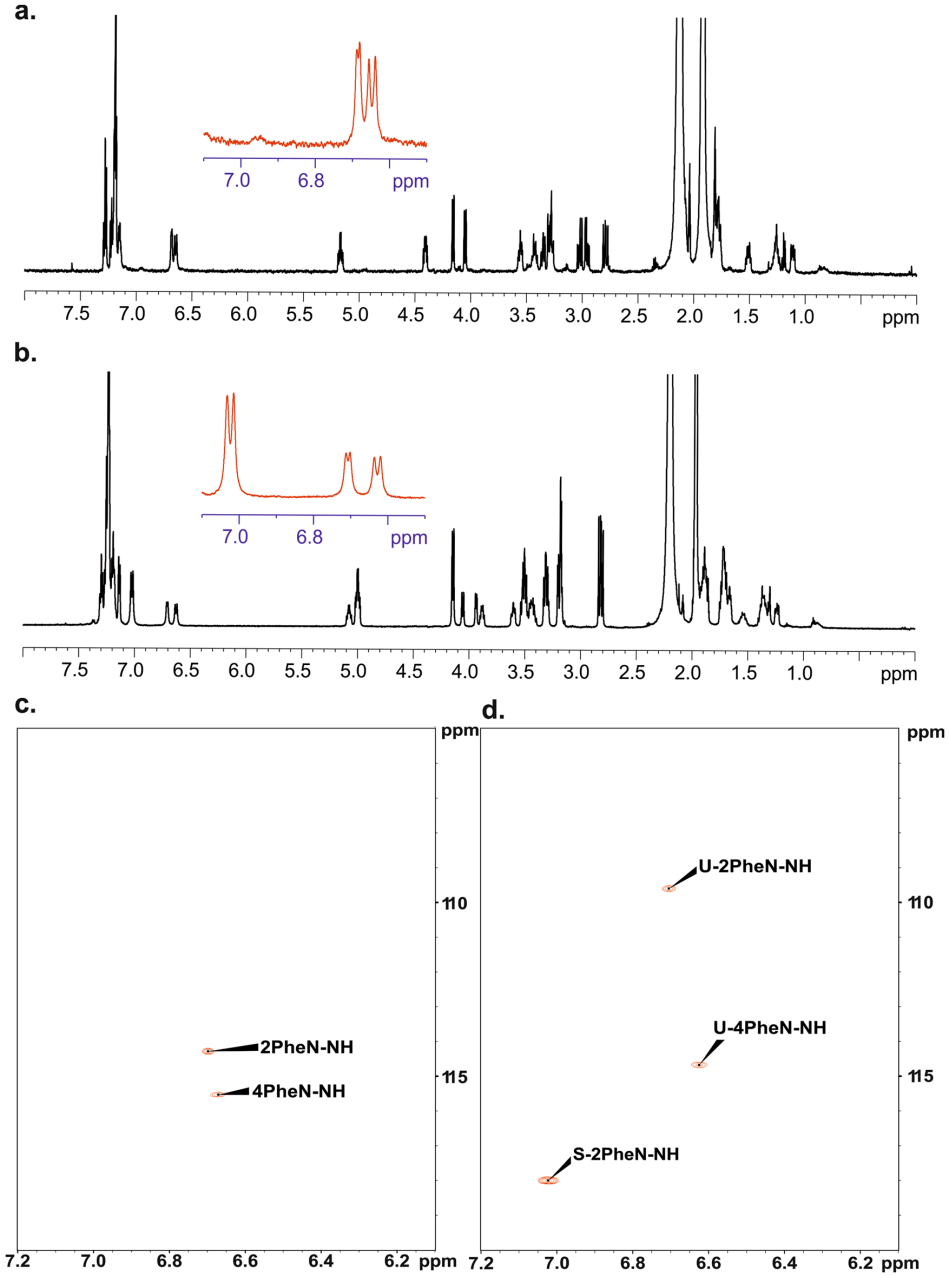
500 MHz ^1^H NMR spectra of sample A (**b**) and sample B (**a**) dissolved in acetonitrile-d_3_. The red inset shown in the middle of the spectrum represents N–H protons. The spectra were recorded at a temperature of 298 K. ^1^H–^15^N HSQC 2D NMR correlations for sample B (**c**) and for sample A (**d**). Sample were dissolved in acetonitrile-d_3_. Symbol S (**d**) represents sample with symmetrical geometry, symbol U represents unsymmetrical sample.

We were interested whether this unexpected effect is only visible in DMSO-d6 (this solvent always contains traces of water) or this phenomenon can also be observed in other solvents. Figure 3a displays ^1^H NMR spectra of samples **A** and **B** recorded in acetonitrile-d3. Analysis of spectra shows that N–H pattern is preserved however chemical shifts of diagnostic protons are significantly different. Thus, for sample **B** these signals are found at δ = 6.68 ppm and δ = 6.65 ppm. The values of vicinal ^3^J_H–H_ coupling constants are equal to 5.01 Hz and 10.90 Hz. For sample **A** we monitored set of three doublets at δ = 6.98 ppm, δ = 6.74 ppm and δ = 6.67 ppm. The vicinal ^3^J_H–H_ coupling constants are found to be 10.26 Hz, 7.07 Hz and 9.91 Hz, respectively. As in previous case the ^15^N chemical shits are different. The values of ^15^N δ_iso_ were taken from ^1^H–^15^N 2D NMR correlations Fig. 3b.

### Variable temperature ^1^H NMR studies of samples A and B dissolved in dimethyl sulfoxide-d_6_ and acetonitryl-d3

**Figure 4:**
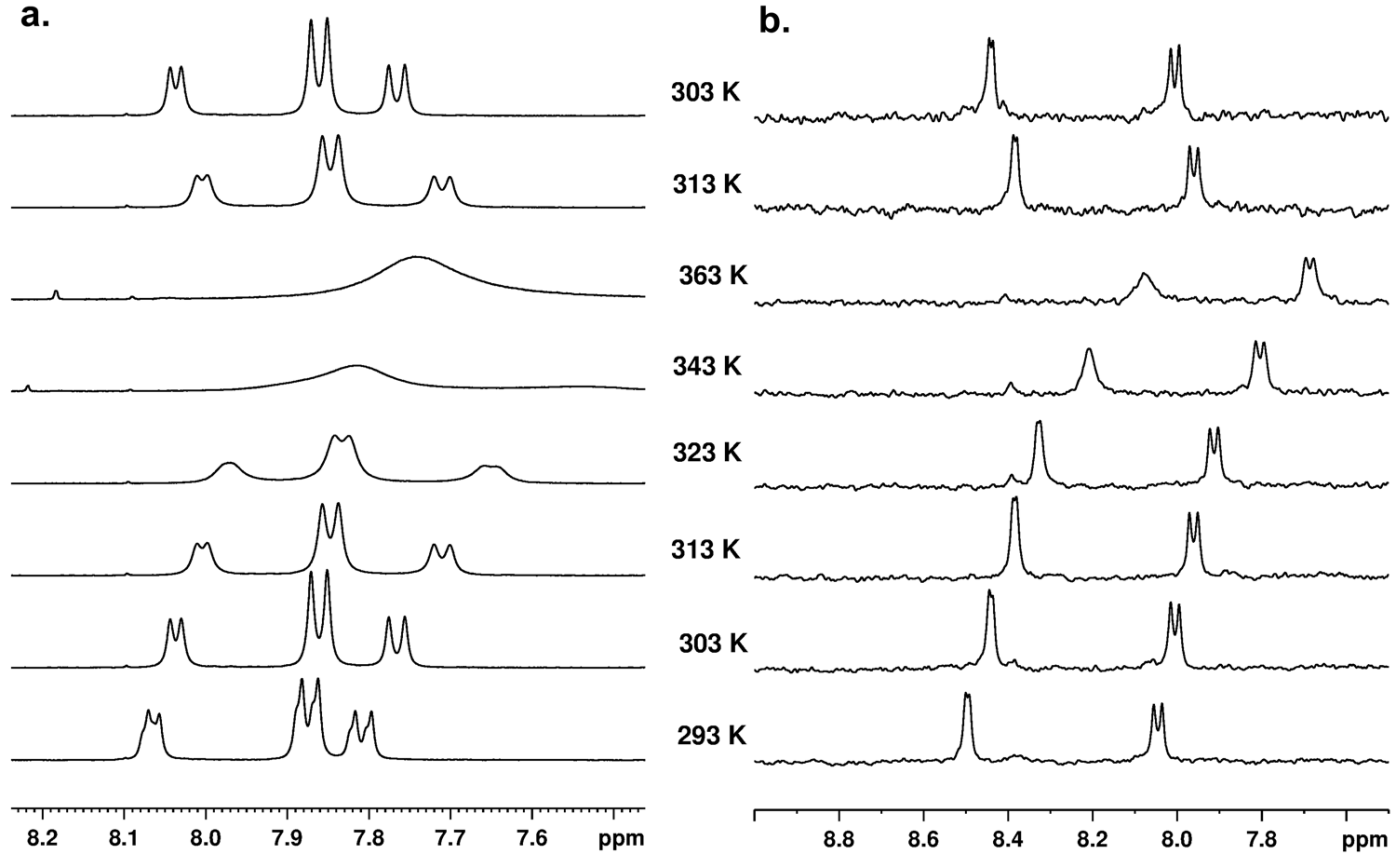
500 MHz ^1^H NMR Variable Temperature (VT) spectra for samples A (left) and B (right) dissolved in DMSO-d6. The measurement temperature is indicated in spectra.

An intriguing and unexpected spectral pattern, particularly for sample **A**, led us to investigate the behaviour of these chemical entities in a function of temperature. Figure 4 shows the ^1^H NMR spectra in region of NH protons. The spectra were recorded in temperature range 293–363 K, samples were dissolved in DMSO-d_6_. Figure 4 (left) shows the ^1^H NMR spectra for sample **A** in range of chemical shift from 8.2 to 7.5 ppm. Spectral analyses show that as the temperature increases, the drift of external signals towards lower values of chemical shift is observed. It is worth noting that the position of the signal, which is between the outer peaks, is rather stable. At 343 K temperature, all peaks are very wide, at temperature of 363 K, the coalescence of signals is observed. When the sample is cooled, the spectral pattern is exactly the same as that of the starting material at the same temperature. This means that the observed changes are reversible. The thermal behaviour of the **B** sample is different. As the temperature increases, a drift of the signals towards lower values of the chemical shift is present, but the coalescence of the NH signals is not observed. As in the previous case with the temperature drop to 303 K, the spectral pattern is the same as for the initial temperature.

**Figure 5:**
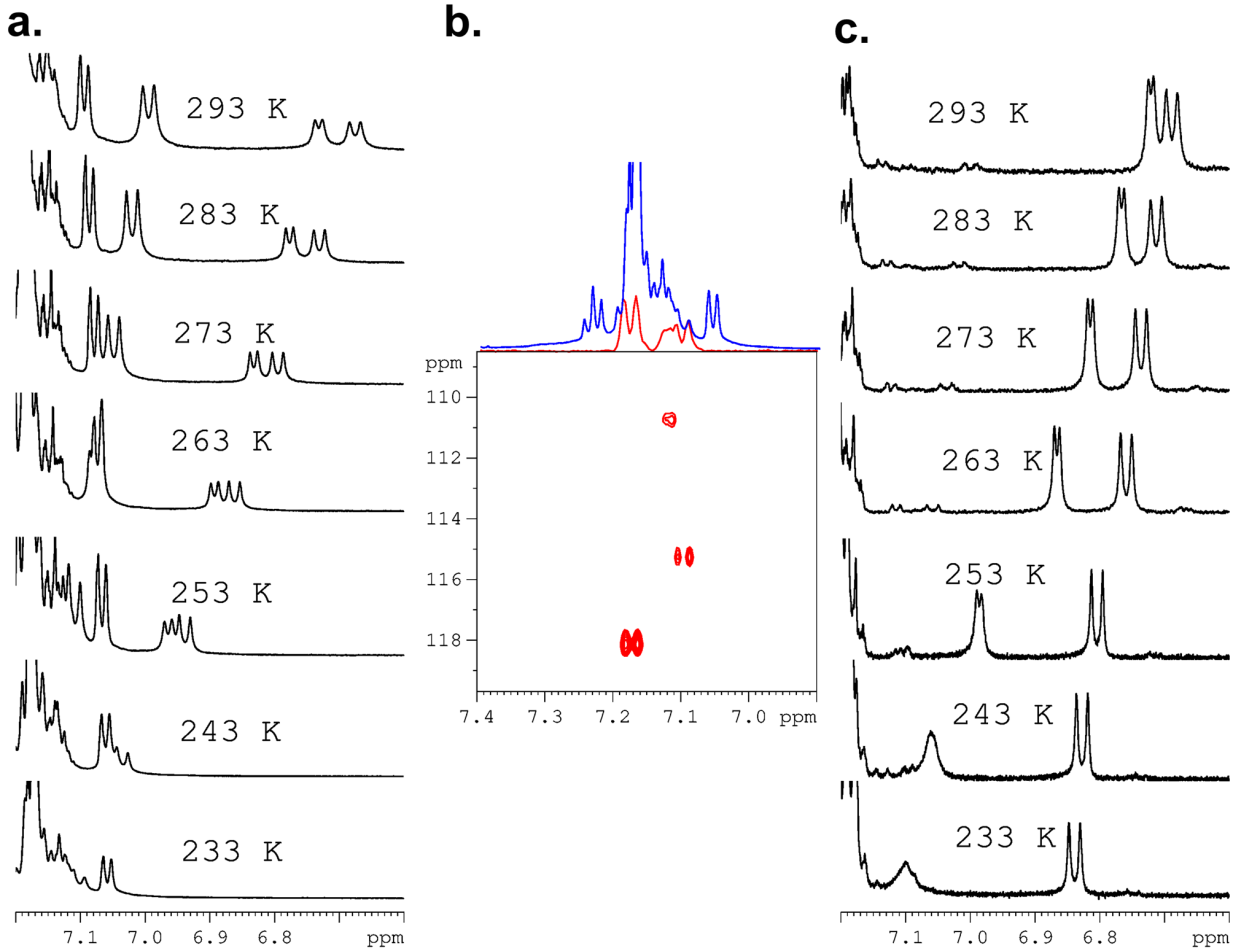
Variable Temperature (VT) NMR spectra for samples dissolved in acetonitrile d_3_. (**a**) ^1^H VT NMR spectra for sample **A**, (**b**) The ^1^H–^15^N HSQC 2D NMR spectrum for sample **A** recorded at 233 K. The F2 projection generated from this correlation is shown as a red spectrum. The blue spectrum represents the ^1^H NMR spectrum recorded at 233 K, as shown in (**a**). (**c**) ^1^H VT NMR spectra for sample **B**. The measurement temperature is indicated in spectra.

In the next step, the thermal properties of samples **A** and **B** dissolved in acetonitrile-d_3_ were investigated. The physicochemical properties of this solvent differ significantly from DMSO-d_6_. The melting point of DMSO-d_6_ is 289 K and that of acetonitrile-d_3_ is 228 K. This feature gives us the ability to control the behaviour of samples at low temperatures. Figure 5a shows the ^1^H VT NMR spectra for sample **A** recorded in temperature range from 293 to 233 K. Spectral inspection shows a shift of NH NMR peaks towards higher values of chemical shifts as the temperature decreases. What's more, as the temperature drops, the NH signals are more “compressed” and the difference between them steadily decreases. At a temperature of 233 K, the NH signals are obscured by the aromatic signals of the phenylalanine residue and are not detectable in practice. Their assignment can be done by means of ^1^H–^15^N HSQC 2D NMR correlation carried out at 233 K (Fig. 5b). It is interesting to note that the difference in the position of NH signals measured as the distance (given in Hz) between the peaks centers at 293 K is equal to 175 Hz, while at 238 K it is 45 Hz. As with the sample dissolved in DMSO-d6, the variable temperature process in acetonitrile-d_3_ is reversible. The behaviour of sample **B** is different compared to sample **A**. First with decrease of the temperature the distance defined as difference of chemical shifts (Δδ) between NH resonances increases. Second, at a temperature of 243 K, the proton signal representing the NH residue belonging to *D*-Phe becomes wide and the J-coupling is not visible. This phenomenon is even more pronounced at temperature of 233 K. There is no simple explanation of this effect. As one of possible explanations we assume that this shifting and line-shape distortion are related with ring current effect. Based on the X-ray structure, we concluded that NH protons are shielded by an aromatic ring and are located in a magnetic shielding cone. It is very likely that as the temperature decreases, the orientation of the phenyl ring in relation to the NH proton changes and this group comes out of the magnetic cone. Moreover we can speculate that aromatic ring undergoes small amplitude wobbling, distance between NH and aromatic ring is not constant what causes dispersion of proton NH signal. In order to study the behavior of the tested samples in other solvents, we carried out measurements in chloroform-d. The ^1^H VT NMR spectra are shown in the supporting materials (see Figure S15).

### Molecular modelling and theoretical calculations of preferred conformations.

The unique and highly complex molecular dynamics processes of cyclic Pro-Phe-Pro-Phe tetrapeptides in the liquid state, stimulated by their differential chirality, have led us to analyze this phenomenon in detail using computational methods. To search for the conformational landscape as a starting point, we used structural models found in the solution of X-ray single crystals (hereinafter referred as X-ray’s). The analysis was performed using Gaussian GMMX software. On the basis of energetic criterium we selected 11 and 9 unique conformers for sample **A** (*LLLL*) and sample **B** (*LLLD*) structure respectively. Table 3 shows the total energies of the systems referenced to the energy for X-ray conformer after DFT calculations. The hetero-chiral system has much larger spread of energy (ca. 80 kJ/mol) compared to homo-chiral system (ca. 16 kJ/mol). However, what is even more important the lowest energy conformer for sample **B** is actually the X-Ray structure, which is not the case for the structure of sample **A**. It implies that the conformation of a homo-chiral system in the liquid state is different to the solid-state conformation.

**Table 3:** Relative conformers energies for sample **A** and **B** after DFT calculations.

Structure ID	Energy (kJ/mol)	
	Sample A	Sample B
X-Ray	0.0	0.0
1	− 4.9	5.6
2	− 4.7	79.7
3	− 6.0	12.4
4	2.1	0.4
5	− 2.6	27.7
6	10.1	18.4
7	4.2	16.8
8	6.0	–
9	− 0.3	–
10	6.0	–

**Figure 6:**
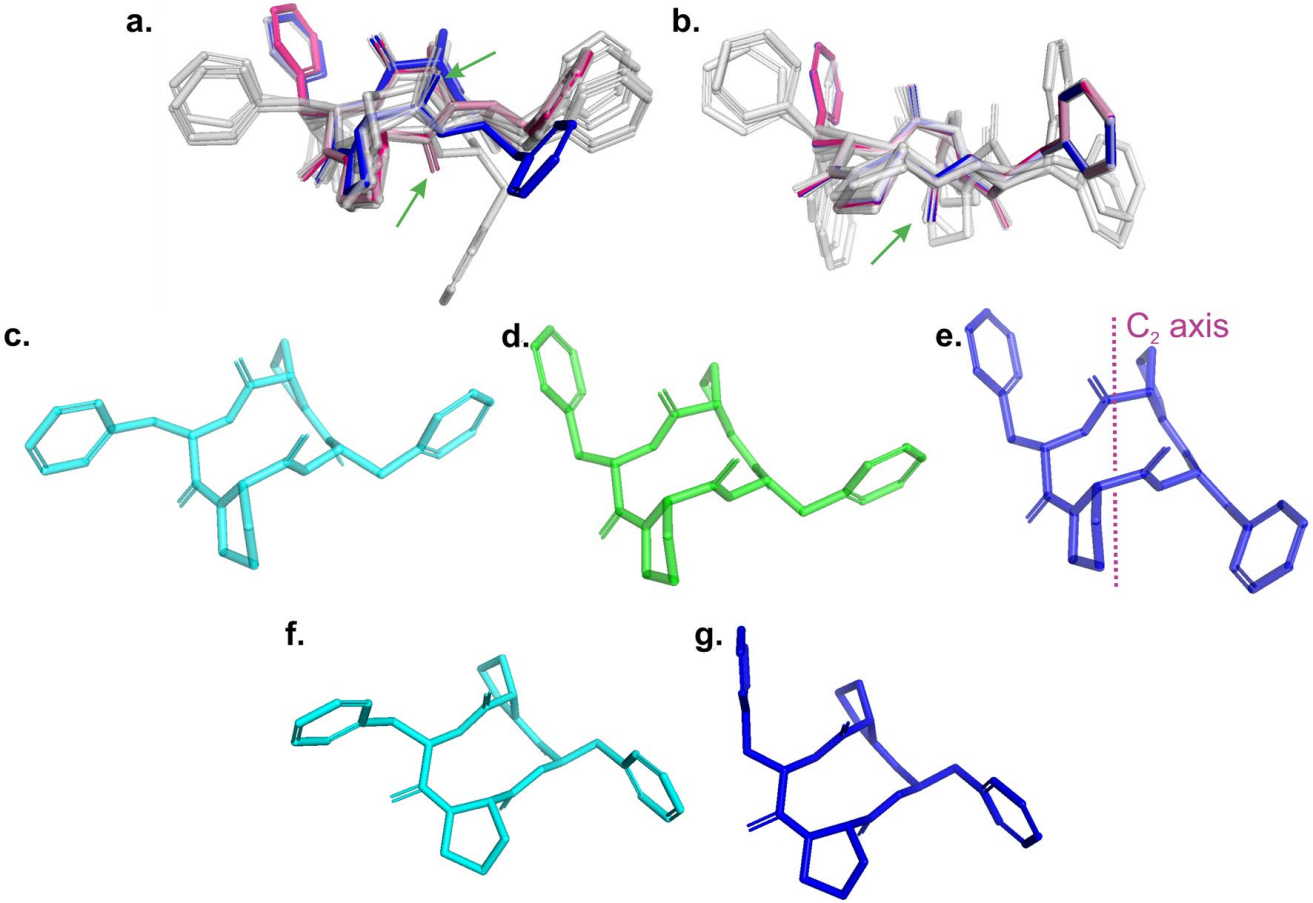
Comparison of all conformers with indicating X-Ray (red) and lowest energy (blue) structure for **A** (**a**) and **B** (**b**). Green arrows indicated the difference in cyclic chain. Comparison of three the lowest energy conformers: (**c**) 1, (**d**) 2 and (**e**) 3 for **A** sample where the structure 3 is the lowest energy conformer and possess C_2_ axis symmetry. Comparison of two the lowest energy conformers: (**f**) 6 and (**g**) X-Ray for **B** sample where the structure X-Ray is the lowest energy conformer. Further details in the main text.

More differences between **A** and **B** systems are visible after the superposition of the conformers (Fig. 6). At first glance, one can see the fundamental difference between X-rays and the structure with the lowest energy (number 3 in Table, yellow segment) indicated by the green arrows for **A** (Fig. 6a). This difference is related to the orientation of the NH and C=O groups of one of the phenylalanine. There is no similar behavior for **B** (Fig. 6b), where all the conformers found have the same peptide bond chain arrangement, including the X-ray conformer which is actually also the lowest energy structure. The three lowest energy structures for **A** are shown in Fig. 6c–e. All of them are about 5 kJ/mol below the energy for the conformer taken from the X-ray solution. In this group the energy for sample 3 is – 6.0 kJ/mol, for 1–4.9 kJ/mol and for 2 is − 4.7 kJ/mol. Different arrangement of the side chains is seen, however, the peptide bond chain is almost the same for all the conformers. An interesting fact is that molecule 3 has a C2 axis symmetry operator, which explains the presence of three sets of NH signals in the proton spectra for **A** and confirms that the two conformers coexist in solution. It is important to note that no restrictive procedures were used in the symmetry calculations.

The lowest-energy for **B** conformers, shown in Fig. 6g, are less surprising. They showed that even such a small energy difference as 0.4 kJ/mol causes quite visible differences in the side chains of the peptide.

In the final stage of this section, we were interested in answering the question of what is the correlation between optimized conformations and the ^1^H NMR spectra. For this reason, we have carried out the DFT calculation in two variants, a sample placed in a vacuum and a sample dissolved in DMSO. In the latter case, we used the PCM model. It is important to note that the PCM model does not consider all the subtle properties of solvents. For example, the possibility of forming hydrogen bonds between the solvent and the investigated medium is omitted. Therefore, in the following paragraphs, we will not discuss the results for acetonitrile because in the calculations we did not observe a significant difference in the NMR parameters for the samples dissolved in these solvents. For the sake of clarity, we will focus on discussing the position of N–H protons, which, as we have shown above, are the most diagnostic elements of the spectra.

**Figure 7:**
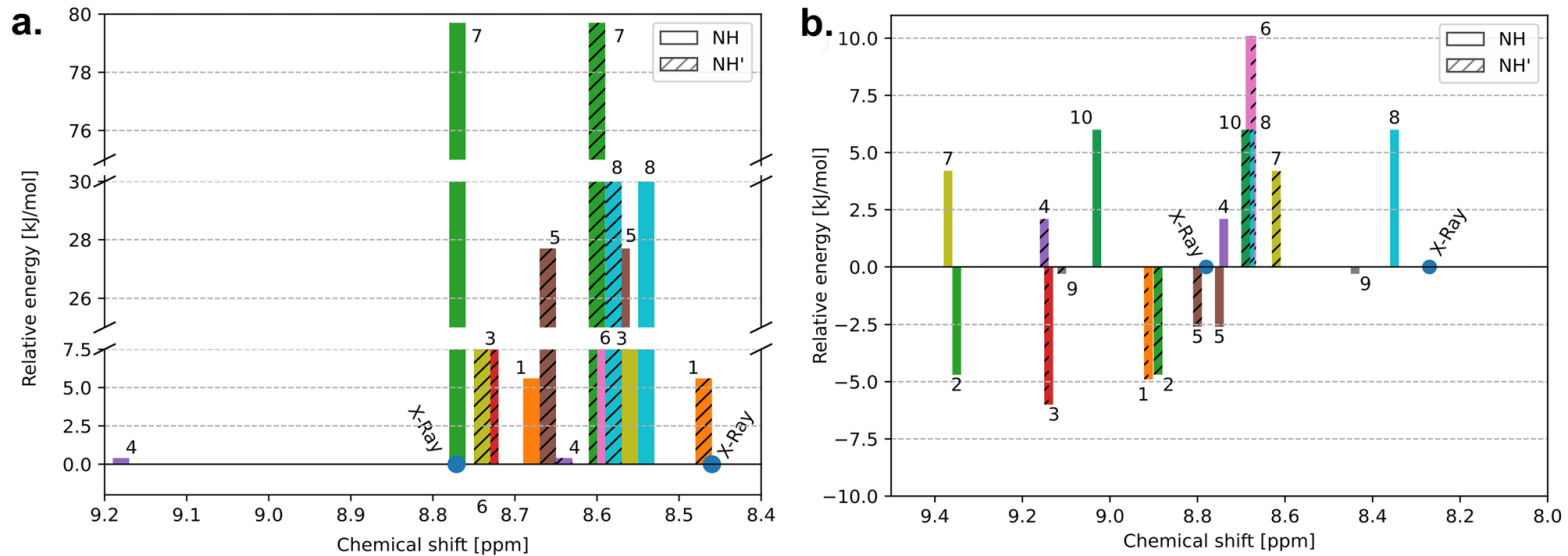
(**a**) Comparison of chemical shifts [ppm] and relative energy [kJ/mol] of conformers for sample A. (**b**) Comparison of chemical shifts [ppm] and relative energy [kJ/mol] of conformers for sample B. The conformation energy of the sample determined from X-ray examinations is arbitrarily set to 0 for A and B independently.

Figures 7a,b shows the relative position of the NH protons, as well as the relative energy for each of the conformers. The conformer energy determined by X-ray examinations is arbitrarily set to 0. The graph is constructed in such a way that sticks of the same colors correspond to the position of NH protons in the same conformer. The analysis of the graph clarifies and confirms the NMR observations presented in “1D and 2D Liquid state NMR studies” section. As we have shown, the **A** structure has three sets of NH signals with an integration ratio of 1:2:1 in the experimental ^1^H spectrum. Figure 7 clearly shows that structure 3 with C2 symmetry has isochronous ^1^H signals, while NH signals for structure 2 are not magnetically equivalent in the liquid state. It is interesting to note that for conformer 1, which has an energy comparable to 2, NH peaks are isochronous, even though this conformer formally lacks C2 symmetry.

Such effects are not observed for sample **B**. First, for each computed conformation the energy is higher compared to X-ray conformation. Second due to broken chirality there is not possible to construct model with C2 symmetry. In each case the two sets of NH protons are observed.

### Solution and solid-state electronic circular dichroism (ECD)

**Figure 8:**
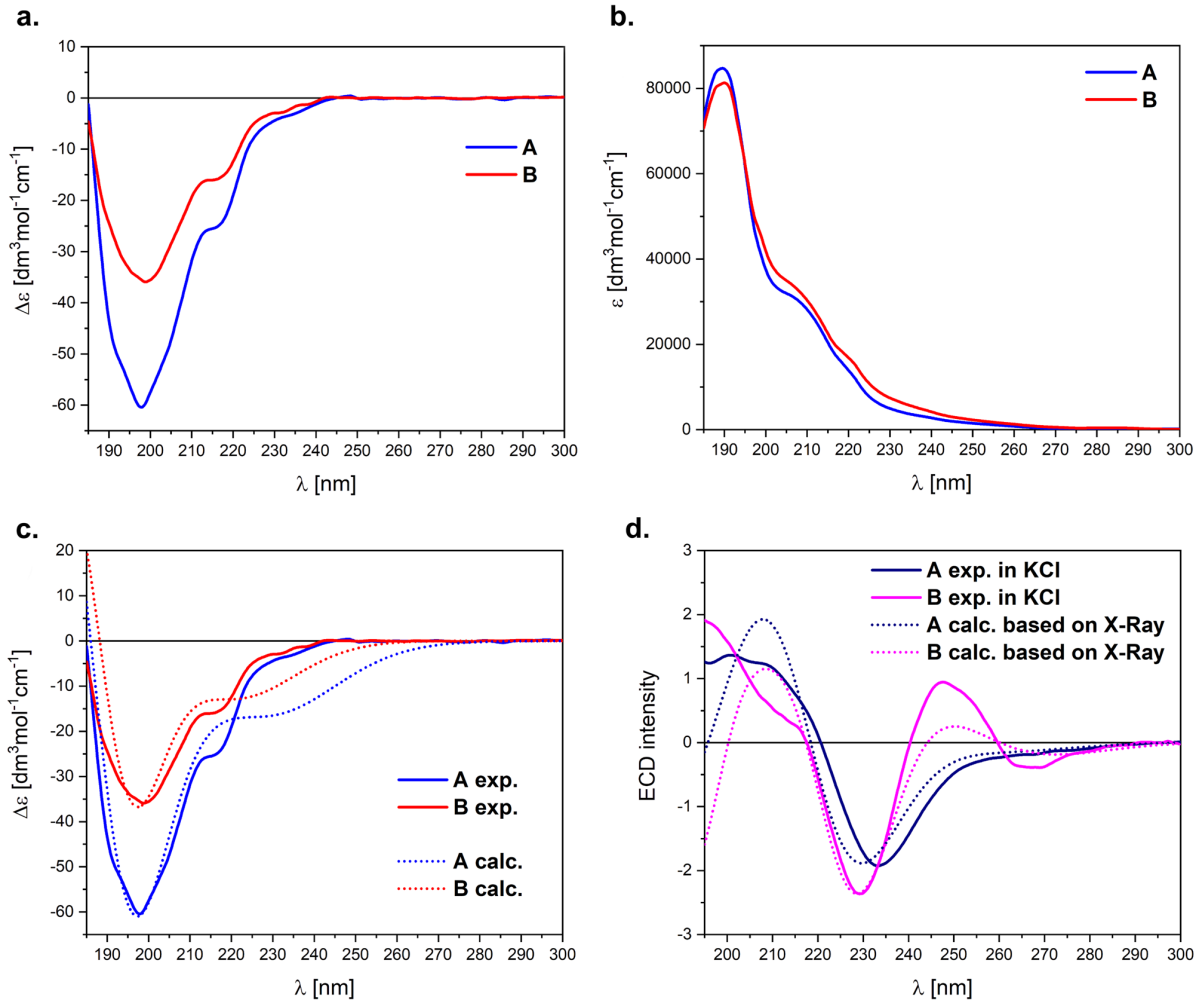
ECD (**a**) and UV (**b**) spectra of **A** and **B** (c = 3.4 × 10^–4^ M) recorded in the mixture CH_3_CN/H_2_O (1:1) at room temperature. (**c**) Solution-phase ECD spectrum of **A** and **B** in comparison with the calculated TDDFT spectra at the ωB97X-D/def2-TZVP/PCM(H_2_O); *Note*: UV-correction = 10 nm, σ = 0.47 eV. (**d**) Solid-state ECD spectra of **A** and **B** in KCl pellet (~ 0.3/100 mg KCl) superimposed with the calculated ECD spectra *in vacuum* based on X-ray structures using TDDFT method at the ωB97X-D/def2-TZVP (after H-optimization); Note: UV-correction = 25 nm, σ = 0.5 eV.

Electronic circular dichroism (ECD) spectroscopy has consistently demonstrated its high utility in examining the configurational and conformational issues in structural chemistry both in solution and solid-state across a diverse range of samples^53^. Its particular sensitivity is seen in study of biomolecules, including, in particular, peptides^54–58^. As can be seen in Fig. 8a,b, the ECD/UV spectra of samples **A** and **B** recorded in the mixture of CH_3_CN/H_2_O (1:1) are similar in the whole spectral range. The only notable difference between ECD spectra lies in the intensity of the negative band(s) centred at ~ 198 nm; ECD spectrum of **A** is ~ 1.7 times more intense. This suggests some structural differences between these compounds, and in fact, above all, they are diastereomers with the opposite absolute configuration in one phenylalanine.

The broad ECD band from 195 to 240 nm is mainly the mixture of n–π* transition of the peptide units with π–π* transition of phenyl groups. In this range there are also two well-pronounced shoulders at ~ 215 and ~ 230 nm. Consequently, the small energy difference between the amide and aromatic excitations makes that the investigated range is very sensitive to any configurational, conformational and environmental differences. Furthermore, in the ECD spectrum there is also a long-wavelength broad band centred at ~ 280 nm related to ^1^L_b_ aromatic excitation of phenyl groups. It intensity is very week, thus is not considered in our solution analysis.

The comparison of experimental ECD spectra with TDDFT calculations provides the inside for conformation equilibrium in solution. Accordingly, in the next step of our investigation, the pool of conformers previously predicted for NMR calculations was used for ECD spectra simulations. The re-optimization was carried out at the B3LYP/6-311 + G(d,p)/PCM(H_2_O) level of theory. The obtained conformations are in line with those predicted for DMSO solution, so that used for analysis of NMR data (see supplementary information, Tables S7 and S8). Finally, the Boltzmann-averaged ECD spectra of the conformers found (> 2%) were calculated at the ωB97X-D/def2-TZVP/PCM(H_2_O) and compared with the experimental ones (Fig. 8c).

According to TDDFT simulations, the ECD spectra of **A** and **B** almost perfectly reproduce the experimental features in solution. The absence of imitation of the shoulder at ~ 215 nm is due to the need to use the same half-width(s) for the entire spectrum; however, it is seen in narrower band-width(s). The pool of computed conformers, although quite different between the investigated samples, precisely reproduces the equilibrium of solution. This further strengthens the conclusion derived from NMR data that **A** is more flexible molecule, and for **B** the lowest energy conformation is one with geometry in solid-state determined by X-ray (Table 3, see Supplementary Information Table S2). On the other hand, it implies that the pool of conformations in both solutions is more varied.

Additionally, we recorded the ECD spectrum for **A** and **B** in solid-state as a KCl microcrystalline pellet (Fig. 8d) to compare the solution and solid-state ECD spectra and to find out the distinctions between the molecular species in the two states of matter. As expected, ECD spectra obtained in KCl pellets are notably different from the measured ones in solution (Fig. 8d), and more importantly they are different from each other.

By applying the solid-state ECD/TDDFT methodology, which consists of calculating the ECD spectrum directly from the X‐ray structure (after optimization of all H-atoms at DFT/B3LYP/6-31G(d) level) we primarily elucidate the chiroptical properties in the solid-state^59^. The results of TDDFT/ECD calculations *in vacuum* at the ωB97X-D/def2-TZVP level of theory are shown also in Fig. 8d. The calculated ECD spectra based on X-Ray structures for **A** and **B** perfectly agree with the experimental ones. On the other hand, no consistency between the solid-state and solution ECD data (Fig. 8c vs. d). pointing out that the geometry in the solid-state determined by the X‐ray analysis (Fig. 6a) is different from the most stable DFT structures in solution equilibrium, which is a consequence of the dynamics processes observed in the solution.

### Molecular dynamics on the base of 2D NMR spectroscopy.

Nuclear Magnetic Resonance (NMR) spectroscopy is a powerful experimental technique for studying molecular motions at the atomic and molecular level. NMR provides valuable information about the dynamics of molecules, including the rates and amplitudes of motions. Several methodological approaches are commonly used depending on the nature and time scale (slow, fast) of molecular movements. Chemical shift anisotropy (CAS) analysis, spin–lattice relaxation time measurements, Carr-Purcell-Meiboom-Gill relaxation dispersion (CPMG), line-shape analysis of deuterium spectra are among the frequently used tools. In our project, we employed a methodology based on the study of dipolar interactions in a liquid state. 2D NMR experiments, such as NOESY, ROESY (Nuclear Overhauser Enhancement Spectroscopy, Rotating Frame Overhauser Enhancement Spectroscopy) or EXSY (EXchange Spectroscopy), can be designed to selectively observe signals from molecules undergoing dynamic processes. These experiments are particularly useful for studying conformational exchange and other dynamic phenomena. The resulting NMR spectrum may contain cross-peaks, which represent correlations between different resonances. These cross-peaks provide information about the exchange between nuclear spins. It is interesting to note that the cross peaks representing dipole interactions and the cross peaks showing molecular dynamics are in opposite phases. In this way, they can be easily recognized and assigned.

**Figure 9:**
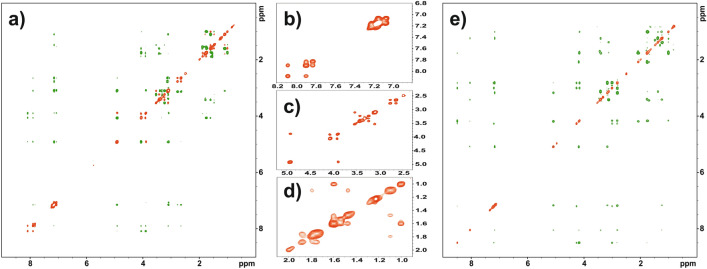
(**a**) ROESY-EXSY 2D NMR spectrum for sample A dissolved in DMSO-d_6_. The green contour-peaks represent the ROESY while the red cross-peaks EXSY correlations, the expanded regions (**b**–**d**) marked in squares. Only EXSY cross-peaks are shown. Spectrum was recorded at temperature 298 K with mixing time equal 300 ms. (**e**) ROESY-EXSY 2D NMR spectrum for sample B dissolved in DMSO-d_6_. The red color represents the diagonal, the EXSY cross peaks are not visible on the spectrum. Spectrum was recorded at temperature 298 K with mixing time equal 300 ms.

Figure 9a shows the 2D ROESY/EXSY spectrum for sample **A** dissolved in DMSO-d_6_, recorded at a temperature of 298 K and a mixing time of 300 ms. EXSY correlation peaks marked in red (ROESY cross peaks are green) are visible in every part of the spectrum. On the right side of the Fig. 9a, the extended part of the spectra marked with squares is displayed. Square b) represents the NH protons and protons of phenylalanine residues for both A conformers. A strong correlation indicates the process of chemical exchange in NH moieties. Analysis of the line-shape of the phenylalanine peaks, located on the diagonal, suggests that the aromatic residues are in a rapid exchange regime. Square c shows the protons CH(alpha) and CH_2_(beta). The in-phase correlation peaks clearly prove that molecular dynamics processes are observed for these residues as well. Finally, the d-square, which represents the protons of proline, allows us to conclude that the five-membered rings undergo rapid conformational exchange on the NMR time scale. In summary, the analysis of the EXSY correlation peaks proves that the homochiral sample **A** is extremely flexible, which is consistent with the theoretical calculations discussed in the previous chapter.

Figure 9b shows the ROESY/EXSY spectrum for a hetero-chiral **B** sample recorded under exactly the same conditions as sample A. DMSO-d_6_ was used as the solvent, the measurement temperature was 298 K, and the mixing time was 300 ms. The measurement results are presented in exactly the same convention as for sample **A**. A comparative analysis of the ROESY/EXSY spectra revealed significant differences between the two samples. The spectrum of the **B** sample is dominated by ROESY correlation peaks. The EXSY cross-peaks representing dynamic processes are quite unique. Such pattern is typical for rigid molecules.

Similar measurements were made for samples **A** and **B** dissolved in acetonitrile-d_3_. The conclusions are very similar. In acetonitrile-d_3_, sample **A** confirmed high flexibility, while sample **B** proved to be stiffer. The appropriate ROESY/EXSY spectra are attached in “Supplementary Information”.

### Interaction of homo-and hetero chiral Pro-Phe-Pro-Phe cyclic peptides with proteins.

As we highlighted in the introduction, cyclic peptides have been used as effective modulators of protein–protein interactions (PPIs). Direct interactions between proteins are an essential subset of the entire network and play a key role in many cellular processes and functions. There are many literature reports supported by deposited X-ray data confirming that cyclic peptides interact with proteins^60–62^.

One of them is the work of Porter and Christianson, who proved that cyclic tetrapeptide trapoxin A containing two phenylalanine residues in the sequence is bound to Class I Histone Deacetylase HDAC8, member of the histone deacetylase family of enzymes^63^. These enzymes play a crucial role in the regulation of gene expression by modifying chromatin structure. HDACs remove acetyl groups from histones, leading to a more condensed and repressive chromatin state, which typically results in the suppression of gene transcription. Dysregulation of HDACs, including HDAC8, has been implicated in various diseases, including cancer and neurological disorders. Abnormal activity of HDACs can lead to the inappropriate silencing of tumour suppressor genes or other critical genes involved in cellular homeostasis. The X-ray structure of histone deacetylase 8 in complex with trapoxin A taken from the Protein Data Bank (PDB ID: 5vi6)^64^ is shown in Supplementary Information (Fig. S16).

One of the conditions that must be met during peptide-protein interactions is the ability of small molecules to adopt a favourable conformation that is preferred at the active site of the enzyme. In this sense, our two cyclic peptides, with very different preferences for conformational changes, appear to be attractive models that can be used to clarify and understand fundamental questions. In order to test the nature of interactions of **A** and **B** with HDAC8, the molecular docking procedure was carried out using GOLD software from the CCDC software package^65^.

**Figure 10:**
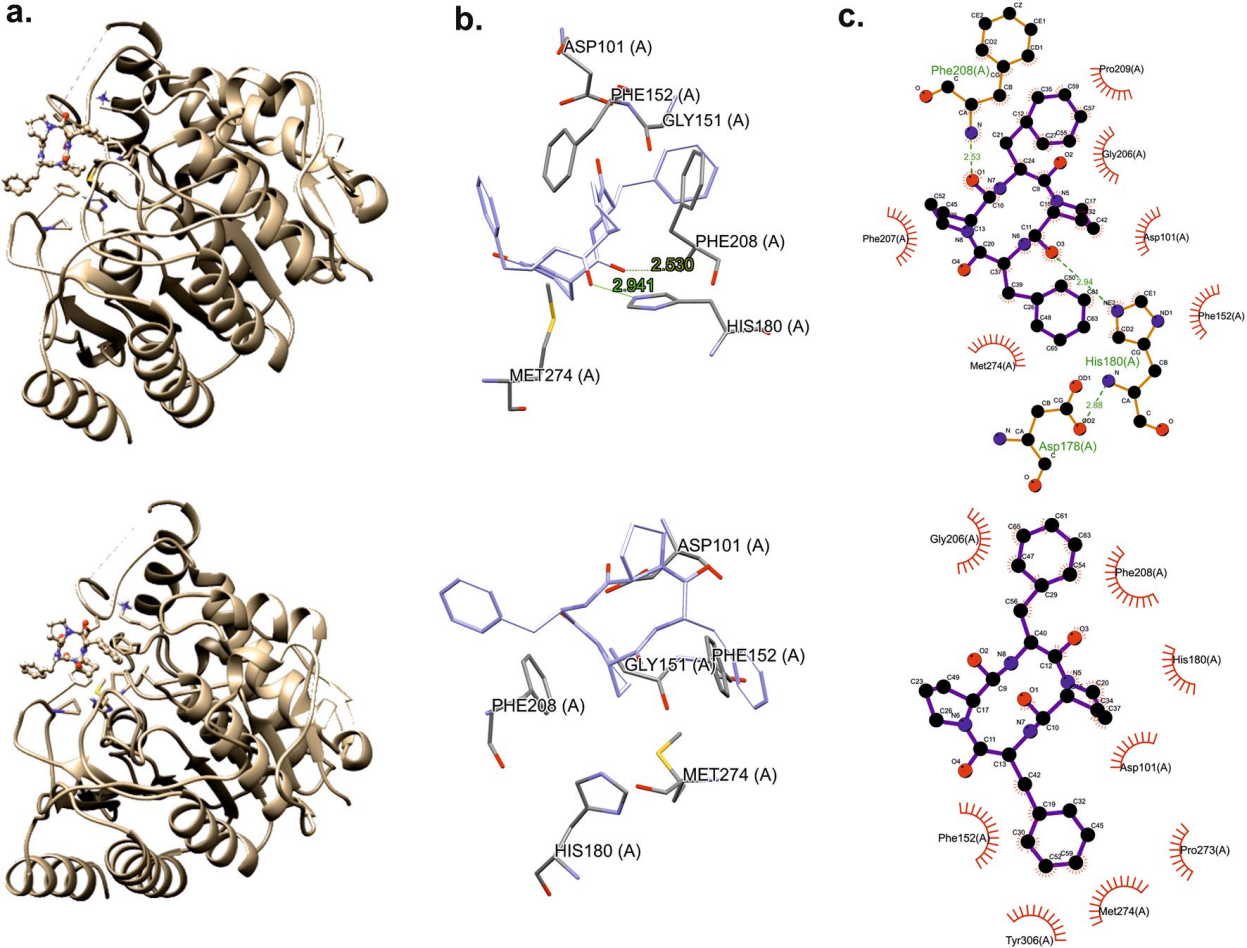
Structures of compounds with docked ligand (**a**). Active site with ligand (**b**), and schematic diagram of the binding (**c**) for sample **A** (top column) and sample **B** (bottom column).

The structure of ligands (A and B) were taken from conformer analysis performed in “Molecular modelling and theoretical calculations of preferred conformations” section. In this step we considered the lowest energy conformation after DFT optimization. Additionally, for the sample A, where the lowest energy conformer is different from X-Ray conformation, we analyzed also X-Ray as well as all other lower energy conformers. The drawings were made using Ligplot+^66,67^, Chimera^68^ and the CCDC software package^69^. Our calculations prove that both compounds bind effectively to the enzyme. The structure and active site are presented in Fig. 10. The docking scoring values for sample **A** were 57.9 (conformer 1), 59.7 (conformer 2), 59.6 (conformer 3) and 58.5 (conformer X-Ray) what prove that the lowest energy conformer bind the protein better than X-Ray conformer.

The results of the molecular docking of ligands are presented in Fig. 10. Molecular docking shows a hydrogen bond and a hydrophobic interaction between the ligand and protein. It is worth noting that the docking scoring value for sample **B** is 50.72, significantly below the values found for sample **A**. This clearly proves that the stereochemistry of even individual amino acid in a sequence has a decisive influence on peptide protein interactions. For both ligands, there are weak hydrogen contacts with the amino acid Asp101 and hydrophobic interaction with the amino acids Met274 and Phe152. Additionally, for the sample **B** compound there are π-π stacking interactions with the Tyr306 amino acid ring. However, in the case of compound **A**, which binds stronger, there are two hydrogen bonds with the amino acids His180 and Phe208 and a π-π stacking interaction with Phe207. These are hydrogen bonds of the N–H….O type.

### Cytotoxicity analysis

**Figure 11:**
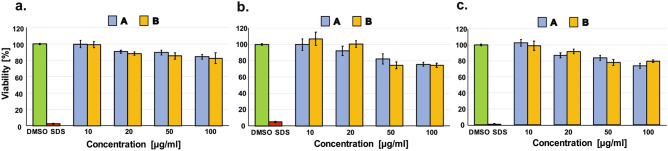
The impact of tested cyclic tetrapeptides on the viability of (**a**) HaCaT, (**b**) A431 and (**c**) MDA-MB-231 cells after 72-h incubation. Results were normalized to cells treated only with DMSO (taken as 100%). SDS was used as a positive control to confirm the validity of the assay. Each bar represents the mean viability value calculated as an average of at least three independent experiments performed in triplicate ± standard error.

In the current work, the cytotoxicity of two diastereomers of cyclic tetrapeptide c(Pro-Phe-Pro-Phe) have been tested to asses a possibility of using these molecules in biological conditions. We have not observed toxic effects against none of the tested human cell types including human keratinocytes HaCaT (Fig. 11a), human epidermoid squamous carcinoma cells (A431) (Fig. 11b) and human breast adenocarcinoma cells (MDA-MB-231) (Fig. 11c) at the concentration range up to 100 µg/ml. The limited toxicity to normal human cells is one of the main prerequisites for considering a potential therapeutic use of drug candidates and testing them in animal models^70,71^. For both tested cyclic peptides, the survival rate of human keratinocytes (HaCaT cells) did not decrease below 80% even after using the compounds at a concentration of 100 µg/ml for 72 h (Fig. 11a). Thus, performed studies have confirmed, that none of the tested cyclic peptide is toxic and both of them might be considered in the context of their possible biological application and might be used for further investigation in biological conditions.

## Conclusions

This paper shows the unique structural features of cyclic c(Pro-Phe-Pro-Phe) tetrapeptides that have never been discussed, although some information regarding this class of compounds has appeared in the literature. Our results revealed that chirality of amino acids has a key influence on formation of conformers and molecular dynamics in the liquid phase. The latter phenomenon is particularly relevant when cyclic peptides are considered to be effective modulators of protein–protein interactions (PPIs). The required ability and agility to adopt the preferred structure in the process of dynamic matching is a characteristic that determines the suitability of the selected compound in biological processes.

In a complementary approach, using X-ray crystallography, circular dichroism, advanced liquid-state NMR spectroscopy, and theoretical calculations, we have clearly demonstrated that a small stereochemical change generates a cascade of unexpected consequences. Analysis of the interaction of cyclic c(Pro-Phe-Pro-Phe) tetrapeptides with the selected enzyme histone deacetylase 8 showed that conformer with all *L* amino acids in sequence is the preferred guest molecule.

## Experimental

### Mechanosynthesis and identification of c(Pro-Phe-Pro-Phe) A and B

All reagents were purchased from Merck Millipore or TCI and used without further purification. The milling treatments were carried out in a vibratory Retsch Mixer Mill MM200.

Tetrapeptide hydrochloride Pro-Phe-Pro-Phe∙HCl (50 mg, 0.09 mmol), TBTU (59 mg, 0.26 mmol, 2.5 eq) and DIPEA (162 µl, 0.9 mmol, 10 eq) were introduced in a 10 mL stainless steel jar with one stainless steel ball (10 mm diameter). The jar was closed and subjected to grinding for 3 h in the vibratory ball mill (MM200) operated at 25 Hz The crude reaction mixture was diluted with CH_2_Cl_2_ (100 mL) and washed with water (2 × 10 mL), 1 M aq. NaHCO_3_ (2 × 10 mL) and brine. The organic layer was dried with MgSO_4_ and concentrated. The yield of the cyclization process, leading to the production of the cyclic products is 72%. The structure of the compounds is confirmed by MS (Fig. S1).

The identification of the reaction products was carried out by ACQUITY UPLC I-Class chromatography system (Waters Corporation, Milford, MA) coupled with SYNAPT G2-Si mass spectrometer equipped with an electrospray ion source and quadrupole-Time-of-Flight mass analyzer (Waters Corp., Milford, MA, USA). For the chromatographic separation of analytes an ACQUITY UPLC™ BEH C18 column (100 × 2.1 mm, 1.7 μm) thermostated at 30 °C temperature was used. Mobile phases consisted of 0.1% formic acid (solvent A) and 0,1% formic acid in acetonitrile (solvent B). A gradient program was developed as follows 15% B (0–0.5 min), 15–70% B (0.5–6.0 min), 70–70% B (6.0–7.0 min), 70–15% B (7.0–7.1 min) and 15–15% B (7.1–9 min). The injection volume was 5 μL and the flow rate was 0.4 mL/min.

For mass spectrometric detection the electrospray source was operated in a positive, high resolution mode. To ensure accurate mass measurements, data were collected in centroid mode. The mass was corrected using leucine enkephalin solution as an external reference (Lock-SprayTM). The optimized source parameters were: capillary voltage 3.05 kV, cone voltage 20 V, source temperature 110 °C, desolvation gas (nitrogen) flow rate 900 L/h with the temperature 350 °C, nebulizer gas pressure 6.5 bar. Mass spectra will be recorded over an *m*/*z* range of 100 to 1200.

Product purity was determined by HPLC (Agilent Technologies 1220 Infinity) using a Pursuit 3 Diphenyl column (150 × 4.6 mm) and UV detector (λ = 220 nm); samples were injected in a volume of 10 μL. The solvent system used for analysis was 0.1% AcOH in water:acetonitrile (80:20, v/v) (solvent A) and 0.1% AcOH in acetonitrile:water (80:20, v/v) (solvent B) at a flow rate of 0.4 ml/min (20–80% B in 45 min).

Homo- and heterochiral c(Pro-Phe-Pro-Phe) **A** and **B** were separated by flash chromatography using Büchi Reveleris X2 system and a cartridge containing a normal stationary phase (Scorpius Silica 40, 60 Å, Irregular, 30 µm) and a mobile phase chloroform (A): methanol (B) at a flow rate of 30 mL/min. Gradient used: isocratic 0% B for 2 min, 0–5% B (2–3 min), 5–5% B (3–5 min), 5–10% B (5–6 min), 10–10% B (6–8 min), 10–15% B (8–9 min), 15–15% B (9–11 min), 15–20% B (11–12 min), 20–20% B (12–14 min), 20–40% B (14–15 min), 40–40% B (15–18 min), 40–90% B (18–19 min), 90–90% B (19–20 min). Cyclic peptides A and B were obtained in yields of 12.2% (6.1 mg) and 6.4% (3.2 mg) respectively, as white solids (Fig. S2).

Product identification and purity after chromatographic separation were determined by HPLC and UPLC-MS, respectively (Figs. S3, S4).

### X-ray diffraction

Single crystals of c(Pro-Phe-Pro-Phe) **A** and c(Pro-Phe-Pro-*d*-Phe) **B** crystallized in methanol were transferred to mineral oil and mounted on cryo loops. The crystals were then flash-cooled directly in a stream of N_2_. Diffraction intensities were recorded using a Rigaku XtaLAB Synergy-S diffractometer equipped with a Cu Kα radiation source (λ = 1.5418 Å) and a HyPix-6000HE hybrid photon counting detector. The total number of runs and images was based on the strategy calculation of the *CrysAlisPro* program (Rigaku, v 1.171.42.96a, 2023). The molecular models of the structures were created with the structure solution program *SHELXT*^72^ using intrinsic phasing with *Olex2*^73^ as the graphical interface and refined by least squares with the 2018/3 version of *SHELXL*^74^. All non-hydrogen atoms were refined anisotropically. The positions of the hydrogen atoms were calculated geometrically and refined using the riding model. The structures were validated with CheckCif (http://checkcif.iucr.org) and deposited in the Cambridge Crystallographic Data Centre (CCDC) under the accession numbers 2330472 and 2330474 for c(Pro-Phe-Pro-Phe) and c(Pro-Phe-Pro-*d*-Phe), respectively.

### ECD spectroscopy

The ECD and UV spectra were recorded using a Jasco J-815 spectrometer (Tokyo, Japan) at room temperature in spectroscopic grade CH_3_CN and distillate water (~ 0.3 mM) mixed 1:1 in quartz cells with a path length ranging from 0.2 to 0.05 cm. The solid-state ECD spectrum (~ 0.3 mg/100 mg KCl) was recorded as a KCl pellet using the methodology described previously^75^. All spectra were measured using a scanning speed of 100 nm min^−1^, a step size of 0.2 nm, a bandwidth of 1 nm, a response time of 0.5 s, and an accumulation of 10 scans. The spectra were background-corrected using spectra of CH_3_CN/H_2_O solvent or empty KCl pellet recorded under the same conditions.

### Theoretical calculations

Conformer search was done in Gaussian GMMX software^76^. Two starting models of **A** and **B** were allowed to vary dihedral angles as well as cartesian coordinates. The Energy window was set to 3.5 kJ/mol and number of trial structures to 10^5^. The applied force field was MMFF94 and MMX^77,78^ as two runs of conformer calculations were done. Only the 13 and 11 conformers were found in the declared energy window for A and B structure respectively. In the following step all the lowest energy conformers including the conformation found in the single crystal X-Ray solution were optimized in Gaussian16^79^ by using B3LYP functional^80,81^ with 6-311 + G(d,p)^82^ basis set and the polarizable continuum model (PCM)^79^ of the solvent. Finally, we got 11 and 9 unique conformers for A and B structure respectively. Calculations of NMR parameters were done in Gaussian16^79^ by using PBE1PBE functional^83,84^ with 6-311++G(d,p)^82^ basis set and the PCM of the solvent for each of individual model.

The ECD spectra were calculated using previously selected set of conformers (see above). All relevant structures were submitted for further DFT re-optimization with Gaussian16^79^ with default grids and convergence criteria using the B3LYP/6-311+G(d,p) level with the PCM solvent model for H_2_O to reflect as best as possible the measurements conditions. The same result was obtained using calculations using PCM for CH_3_CN. All DFT conformers were confirmed to contain no imaginary frequencies. The final pool of conformers for simulations of chiroptical properties was identified within 3 kcal/mol. ECD/UV calculations were run at the TDDFT level with the ωB97X-D functional and the def2-TZVP basis set in the PCM model for H_2_O. The CAM-B3LYP and B3LYP was also tested; CAM-B3LYP gave very similar results with ωB97X-D, while B3LYP gave worse results in comparison to the experimental data. Average ECD/UV spectra were computed by weighting spectra of individual conformers using Boltzmann factors at 298 K estimated beforehand from DFT internal energies. All conformers having population ≥ 3% at 298 K were taken into consideration. The final spectra were generated using the programme SpecDis ver. 1.70. Solid-state ECD spectra were calculated using the obtained X-ray structures for **A** and **B** owing to the methodology described in Ref.^75^.

### NMR spectroscopy

Measurements were performed on Bruker AV III 500 spectrometer operating at 500.13 and 50.67 MHz for ^1^H and ^15^N, respectively using 5 mm TBO (BB-H/F-D) probehead with z-gradients coil and GAB/2 gradient unit capable to produce B0 gradients with maximum strength of 50 G/cm. The BCU-05 cooling unit controlled by VTU3200 temperature unit was used for temperature regulation and stabilization. Bruker TopSpin 3.5 program was used for controlling the acquisition and processing of the spectra.

For ^1^H spectra usually 128 scans were acquired with relaxation delay of 1 s and other parameters set as follow: TD = 64 k, SW = 24.5 ppm (12,335 Hz), AQ = 2.65 s. For variable temperature measurements the sample was thermostated at the desired temperature for 5 min before acquisition.

^1^H–^15^N HSQC spectra were recorded in 2 K × 256 (F2 × F1) data point matrix with relaxation delay of 1.5 s with Bruker original pulse program. Usually, 64 or 128 scans were collected per experiment. Spectral width (SW) was set to 11 ppm (5500 Hz) and 100 ppm (5068 Hz) for ^1^H(F2) and ^15^N (F1), respectively. Prior to Fourier transformation, the FIDs were apodized with a QSINE (SSB = 2) function in both dimensions. Final data had size of 4 K × 1 K (F2xF1) data points.

### Cytotoxicity evaluation

*Cell culture*. The cytotoxicity experiments were performed using two cancer cell lines: human epidermoid squamous carcinoma cells (A431) and human breast adenocarcinoma cells (MDA-MB-231), as well as non-cancerous human epidermal keratinocytes (HaCaT). The A431 and HaCaT cells were cultured in a medium consisting of Dulbecco's Modified Eagle Medium (DMEM) (Sigma-Aldrich, St. Louis, MO, USA), 10% Fetal Bovine Serum (FBS) (v/v) (Sigma-Aldrich, St. Louis, MO, USA) and Penicillin–Streptomycin solution (10000 U/mL) (Sigma-Aldrich, St. Louis, MO, USA). In case of MDA-MB-231 cells, medium was supplemented with L-glutamine and MEM non-essential amino acid solution (Sigma-Aldrich, St. Louis, MO, USA). The cell cultures were carried out at 37 °C in the atmosphere supplemented with 5% CO_2_.

*Cell Viability Assay*. The MTT cell viability assay was used to evaluate the cytotoxicity of the studied compounds. The compounds were dissolved in DMSO to get concentrations of 1, 2, 5, 10 mg/ml. The MTT test is based on the conversion of a water-soluble tetrazolium salt (3-(4,5-dimethylthiazol-2-yl)-2,5-diphenyltetrazolium bromide) into insoluble formazan by the mitochondrial dehydrogenase enzyme. After dissolving the formazan crystals in isopropanol, a colored solution was measured for the absorbance intensity which is proportional to the number of metabolically active cells.

Cells were plated at 10,000 per well in a 96-well plate and incubated overnight under standard conditions (37 °C and 5% CO_2_). Then, the used culture medium was removed and 100 µl of the fresh one, containing tested compounds was added, and incubated for 72 h. The solution of DMSO (at 1% final concentration) was also used as a negative control and SDS solution (at 2% final concentration) as a positive control. Cells were incubated with tested compounds for 72 h. After incubation period, 20 µl of MTT solution was added and the cells were incubated for another 2 h under standard conditions (37 °C and 5% CO_2_). After that the medium was removed and 100 µl isopropanol was added to each well and mixed by shaking at room temperature until the crystals were dissolved. The absorbance level was measured at 570 nm with 630 nm as a reference using a Synergy HT plate reader (BioTek Instruments, Inc, Winooski, Vermont, USA) and KC4 3.2 Rev. 2 software (BioTek Instruments, Inc, Winooski, Vermont, USA).

### Supplementary Information

Supplementary Information.

## Data Availability

All data generated or analysed during this study are included in this published article and its supplementary information files.
