# Supplementary material for: Influence of heterochirality on the structure, dynamics, biological properties of cyclic(PFPF) tetrapeptides obtained by solvent-free ball mill mechanosynthesis
Source: Sci Rep. 2024 Jun 4;14:12825. doi: 10.1038/s41598-024-63552-4 (PMC11150477; doi:10.1038/s41598-024-63552-4)
Supplement: Supplementary file 1 — Supplementary Information. [file 41598_2024_63552_MOESM1_ESM.pdf]

Supplementary information

Table of Contents

1. Mechanosynthesis and identification of c-PFPF A and B **2**

Figure S1. UPLC-ESI(+)-MS chromatograms of Pro-Phe-Pro-Phe·HCl with TBTU and DIPEA after 3h of milling and washed with water. a) total ion chromatogram (TIC), b) extracted ion chromatogram (XIC) of monoisotopic peak of cyclic tetrapeptide cPro-Phe-Pro-Phe (*m/z* 489.2502). 2

Figure S2. Flash chromatogram of the crude mixture after the reaction of ball grinding·for 3 hours of Pro-Phe-Pro-Phe·HCl with TBTU and DIPEA and washing with water. 2

Figure S3. HPLC chromatograms of of separated cyclic tetrapeptides c(Pro-Phe-Pro-Phe). a) c(Pro-Phe-Pro-Phe) and b) c(Pro-Phe-Pro-*D*-Phe). 3

Figure S4. Total ion chromatograms (TIC) and ESI(+)-MS spectra of separated cyclic tetrapeptides c(Pro-Phe-Pro-Phe). a) c(Pro-Phe-Pro-Phe) and b) c(Pro-Phe-Pro-*D*-Phe). 3

2. Structural data from scXRD **3**

Table S1: Fractional Atomic Coordinates (×10^4) and Equivalent Isotropic Displacement Parameters (Å^2×10^3) for **c(Pro-Phe-Pro-Phe)** and **c(Pro-Phe-Pro-D-Phe)**, respectively. U_{eq} is defined as 1/3 of the trace of the orthogonalized U_{ij}. 3

Table S2: Anisotropic Displacement Parameters (×10^4) for **c(Pro-Phe-Pro-Phe)** and **c(Pro-Phe-Pro-D-Phe)**, respectively. The anisotropic displacement factor exponent takes the form: $-2\pi^2[h^2a^{*2} \times U_{11} + ... +2hka^* \times b^* \times U_{12}]$ 4

Table S3: Bond Lengths in Å for **c(Pro-Phe-Pro-Phe)** and **c(Pro-Phe-Pro-D-Phe)**, respectively. 5

Table S4: Bond Angles in for **c(Pro-Phe-Pro-Phe)** and **c(Pro-Phe-Pro-D-Phe)**, respectively. 5

Table S5: Torsion Angles in for **c(Pro-Phe-Pro-Phe)** and **c(Pro-Phe-Pro-D-Phe)**, respectively. 6

Table S6: Hydrogen Fractional Atomic Coordinates (×10^4) and Equivalent Isotropic Displacement Parameters (Å^2×10^3) for **c(Pro-Phe-Pro-Phe)** and **c(Pro-Phe-Pro-D-Phe)**, respectively. U_{eq} is defined as 1/3 of the trace of the orthogonalized U_{ij}. 8

Figure S5. A crystal packing diagram of (*a*) c(Pro-Phe-Pro-Phe) (sample A) and (*b*) c(Pro-Phe-Pro-D-Phe) (sample B). The views along the *b*-axis are shown above, while the views along the *a*-axis are shown below. 9

3. Theoretical Calculations and ECD Spectroscopy **9**

Table S7. Relative conformers energies with population at 298 K for sample **A** after DFT calculations at the B3LYP/6-311+G(d,p)/PCM(H_2O) level of theory. 9

Table S8. Relative conformers energies with population at 298 K for sample **B** after DFT calculations at the B3LYP/6 311+G(d,p)/PCM(H_2O) level of theory. 9

4. Molecular dynamics on the base of 1D and 2D NMR spectroscopy **10**

Table S9. cPFPF sample heteriochiral B. 10

Figure S7. NMR TOCSY ^{1}H-^{1}H spectrum of cPFPF-B in DMSO-d6 (Heterochiral). 11

Figure S8. NMR spectra HSQC ^{1}H-^{15}N (a) and HSQC ^{1}H-^{13}C (b) of cPFPF-B in CD_3CN (Heterochiral). 11

Figure S10. NMR NOESY ^{1}H-^{1}H spectrum of cPFPF-B, CD_3CN (Heterochiral). 13

Table S10. NMR chemical shifts of cPFPF sample homochiral A. 13

Figure S11-14. NMR HSQC ^{1}H-^{15}N (a) and HSQC ^{1}H-^{13}C (b) spectra of cPFPF-A, CD_3CN (Homochiral). Prefix S means form of peptide A with 2-fold symmetry and prefix U means unsymmetrical form of A. 14

Figure 15. 600 MHz ^{1}H NMR spectra of N-H region of sample A and sample B dissolved in chloroform-d. 16

Figure S16. Crystal structure of histone deacetylase 8 in complex with trapoxin A. 17

1. Mechanosynthesis and identification of c-PFPF A and B

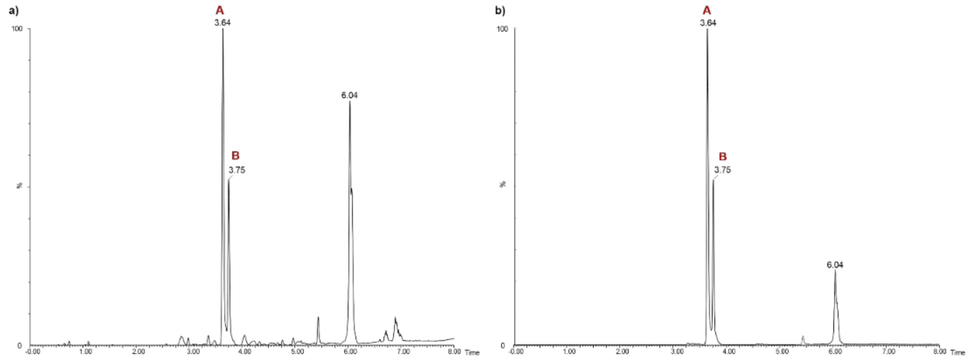

Figure S1. UPLC-ESI(+)-MS chromatograms of Pro-Phe-Pro-Phe·HCl with TBTU and DIPEA after 3h of milling and washed with water. a) total ion chromatogram (TIC), b) extracted ion chromatogram (XIC) of monoisotopic peak of cyclic tetrapeptide cPro-Phe-Pro-Phe (*m/z* 489.2502).

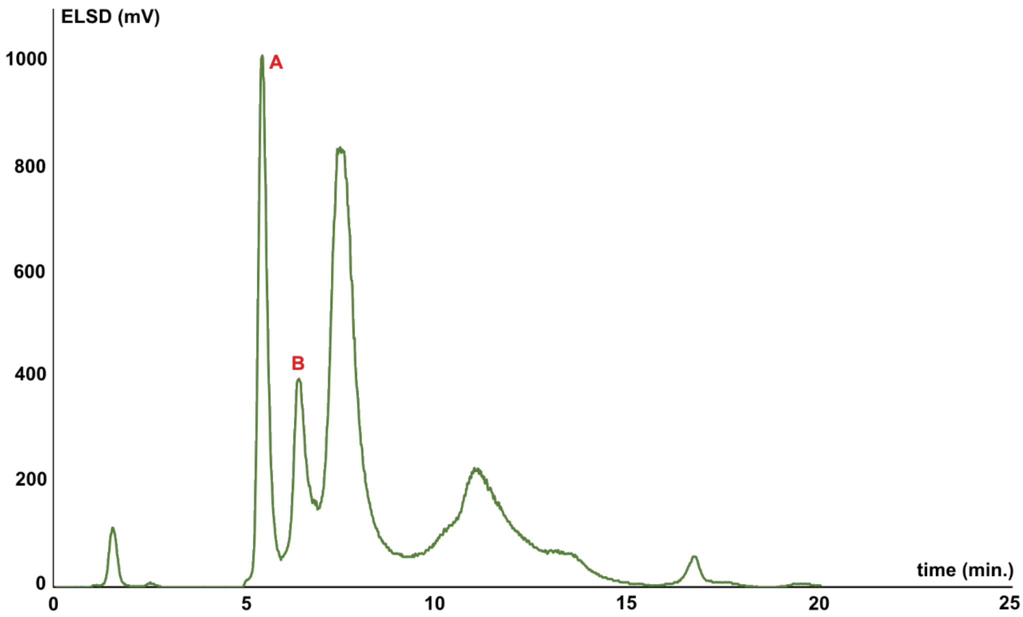

Figure S2. Flash chromatogram of the crude mixture after the reaction of ball grinding·for 3 hours of Pro-Phe-Pro-Phe·HCl with TBTU and DIPEA and washing with water.

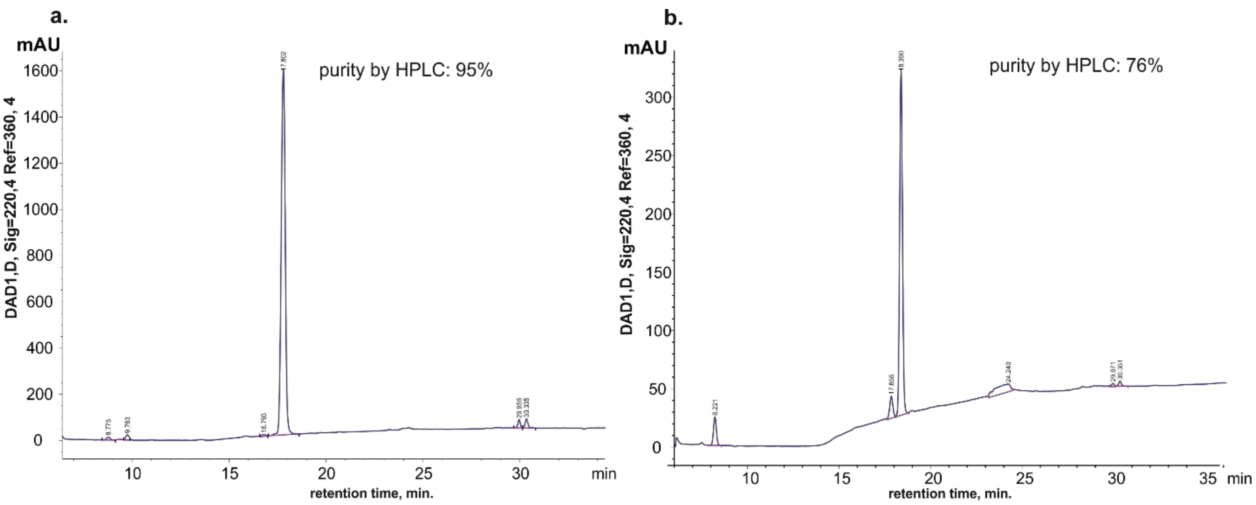

Figure S3. HPLC chromatograms of of separated cyclic tetrapeptides c(Pro-Phe-Pro-Phe). a) c(Pro-Phe-Pro-Phe) and b) c(Pro-Phe-Pro-*D*-Phe).

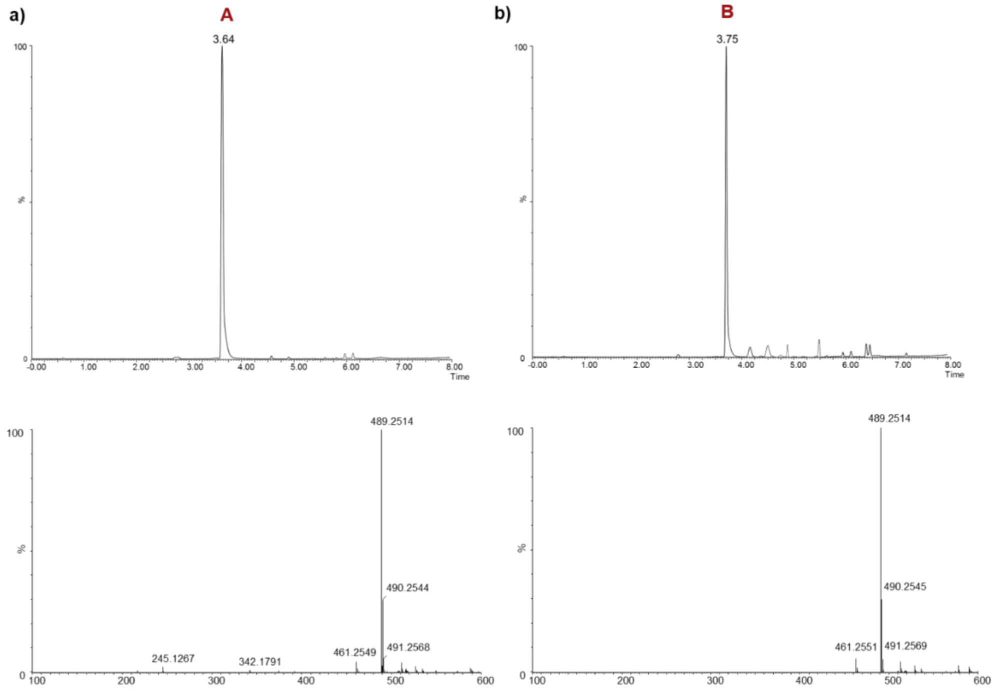

Figure S4. Total ion chromatograms (TIC) and ESI(+)-MS spectra of separated cyclic tetrapeptides c(Pro-Phe-Pro-Phe). a) c(Pro-Phe-Pro-Phe) and b) c(Pro-Phe-Pro-*D*-Phe).

2. Structural data from scXRD

Table S1: Fractional Atomic Coordinates (×10^4) and Equivalent Isotropic Displacement Parameters (Å^2×10^3) for **c(Pro-Phe-Pro-Phe)** and **c(Pro-Phe-Pro-D-Phe)**, respectively. U_{eq} is defined as 1/3 of the trace of the orthogonalized U_{ij}.

c(Pro-Phe-Pro-Phe)					c(Pro-Phe-Pro-D-Phe)				
**Atom**	***x***	***y***	***z***	U_{eq}	**Atom**	***x***	***y***	***z***	U_{eq}
**N1**	5582.2(13)	6376(4)	1285.4(9)	14.8(4)	**N1**	4878.3(18)	5951(5)	3761.4(12)	18.7(5)
**C2**	6058.1(15)	4203(5)	1751.7(11)	14.0(4)	**C2**	4331(2)	3750(6)	3299.4(14)	19.0(6)
**C3**	6101.0(15)	4276(5)	2630.1(11)	14.3(4)	**C3**	4070(2)	3840(6)	2432.4(15)	18.1(6)
**O4**	6176.9(12)	2236(3)	2974.3(9)	19.5(4)	**O4**	3937.9(16)	1769(4)	2103.1(11)	23.2(5)
**C5**	7209.2(16)	4174(5)	1617.1(12)	19.3(5)	**C5**	3222(2)	3600(6)	3471.6(15)	23.5(6)
**C6**	7407.3(17)	6948(5)	1443.9(12)	20.7(5)	**C6**	3031(2)	6336(7)	3683.2(16)	25.1(7)
**C7**	6367.4(17)	7734(5)	930.5(12)	18.0(5)	**C7**	4162(2)	7136(6)	4162.3(15)	21.7(6)
**N8**	6098.5(13)	6532(4)	2982.5(10)	14.3(4)	**N8**	3939.0(18)	6095(5)	2074.3(13)	17.9(5)
**C9**	6279.6(16)	6735(5)	3831.3(11)	15.3(5)	**C9**	3527(2)	6262(6)	1242.4(14)	19.7(6)
**C10**	5452.1(17)	8372(5)	4121.9(11)	17.2(5)	**C10**	4240(2)	7928(6)	895.7(15)	20.8(6)
**O11**	5727.4(13)	9914(3)	4648.8(9)	23.1(4)	**O11**	3815.2(17)	9529(4)	410.7(11)	27.3(5)
**C12**	7376.7(17)	7856(5)	4166.7(12)	20.0(5)	**C12**	2357(2)	7305(6)	982.2(16)	24.4(6)
**C13**	8295.6(16)	6388(5)	3980.5(12)	19.3(5)	**C13**	1520(2)	5666(6)	1183.3(15)	22.6(6)
**C14**	8632.9(18)	4134(5)	4362.9(12)	23.0(5)	**C14**	1089(2)	6393(7)	1759.7(16)	28.0(7)
**C15**	8870.5(19)	7355(5)	3456.1(13)	24.2(5)	**C15**	1114(2)	3476(7)	774.0(16)	26.7(7)
**C16**	9533(2)	2898(6)	4237.1(15)	31.7(6)	**C16**	278(3)	4950(7)	1917.1(19)	34.1(8)
**C17**	9769(2)	6117(6)	3328.5(16)	34.3(7)	**C17**	295(3)	2043(7)	926.7(18)	32.5(8)
**C18**	10103.4(19)	3905(7)	3720.0(17)	36.2(7)	**C18**	-124(3)	2785(8)	1498(2)	35.1(8)
**N19**	4418.9(14)	8041(4)	3801.0(10)	16.6(4)	**N19**	5324.8(18)	7498(5)	1098.3(12)	20.5(5)
**C20**	3950.1(16)	5964(5)	3299.8(12)	15.2(5)	**C20**	5953(2)	5589(6)	1622.1(15)	20.2(6)
**C21**	3879.9(15)	6584(4)	2435.1(11)	13.7(4)	**C21**	6210(2)	6454(6)	2444.4(15)	18.9(6)
**O22**	3822.5(11)	8754(3)	2181.1(8)	17.8(4)	**O22**	6269.5(15)	8710(4)	2630.8(11)	24.2(5)
**C23**	2819.9(17)	5815(5)	3464.0(12)	19.3(5)	**C23**	7034(2)	5463(6)	1418.1(16)	24.7(7)

	c(Pro-Phe-Pro-Phe)					c(Pro-Phe-Pro-*D*-Phe)			
Atom	*x*	*y*	*z*	U_{eq}	Atom	*x*	*y*	*z*	U_{eq}
**C24**	2586.9(17)	8584(5)	3636.0(12)	21.2(5)	**C24**	7161(2)	8199(6)	1158.9(17)	27.4(7)
**C25**	3632.2(17)	9436(5)	4137.8(12)	19.0(5)	**C25**	5994(2)	8959(7)	722.0(16)	24.6(6)
**N26**	3867.9(14)	4509(4)	1983.9(10)	14.4(4)	**N26**	6419.5(19)	4536(6)	2954.2(14)	20.3(5)
**C27**	3742.8(15)	4729(4)	1140.6(11)	14.4(5)	**C27**	6823(2)	5134(7)	3758.8(16)	24.4(7)
**C28**	4553.9(16)	6550(5)	907.3(11)	15.7(5)	**C28**	5968(2)	6237(6)	4099.8(15)	20.7(6)
**O29**	4284.9(12)	8087(3)	377.8(8)	20.9(4)	**O29**	6318.5(16)	7555(5)	4670.1(11)	27.1(5)
**C30**	2625.3(16)	5588(5)	753.3(12)	17.5(5)	**C30**	7482(2)	2936(7)	4207.4(16)	27.6(7)
**C31**	1758.9(16)	3870(5)	907.8(11)	17.1(5)	**C31**	8559(2)	2665(7)	4030.5(16)	26.7(7)
**C32**	1158.5(17)	4521(6)	1457.5(12)	23.4(5)	**C32**	8698(2)	800(7)	3542.1(18)	31.3(7)
**C33**	1508.8(17)	1648(5)	484.4(13)	20.2(5)	**C33**	9416(3)	4306(7)	4358.8(18)	34.6(8)
**C34**	328.8(19)	2990(6)	1570.7(15)	29.6(6)	**C34**	9698(3)	522(8)	3399(2)	40.9(9)
**C35**	678.1(18)	122(5)	593.6(14)	25.2(5)	**C35**	10394(3)	4074(9)	4206.4(19)	42.1(9)
**C36**	86.7(18)	795(6)	1139.8(16)	29.8(6)	**C36**	10551(3)	2196(9)	3735(2)	43.8(10)

Table S2: Anisotropic Displacement Parameters (×10^4) for **c(Pro-Phe-Pro-Phe)** and **c(Pro-Phe-Pro-*D*-Phe)**, respectively. The anisotropic displacement factor exponent takes the form: $-2\pi^2[h^2a^{*2} \times U_{11} + ... +2hka^* \times b^* \times U_{12}]$

	c(Pro-Phe-Pro-Phe)						c(Pro-Phe-Pro-*D*-Phe)					
Atom	U_{11}	U_{22}	U_{33}	U_{23}	U_{13}	U_{12}	Atom	U_{11}	U_{22}	U_{33}	U_{23}	U_{13}	U_{12}
**N1**	19.9(8)	9.7(11)	15.5(7)	1.1(7)	5.4(6)	-2.8(7)	**N1**	22.9(10)	14.9(14)	19.8(10)	-2.2(10)	8.7(9)	1.3(10)
**C2**	18.1(9)	8.2(12)	16.3(9)	-0.2(9)	4.6(7)	-0.3(8)	**C2**	22.7(12)	14.0(16)	21.6(12)	0.7(12)	8.5(10)	-0.7(11)
**C3**	14.7(8)	10.5(13)	17.6(9)	-1.0(9)	3.0(7)	0.2(8)	**C3**	18.1(11)	12.9(16)	25.8(13)	0.1(12)	10.5(10)	0.1(11)
**O4**	28.8(8)	8.8(10)	20.0(7)	1.9(6)	3.0(6)	0.4(7)	**O4**	32.0(10)	13.0(12)	25.2(10)	-1.1(8)	10.0(8)	-0.1(9)
**C5**	17.6(9)	19.9(14)	21.1(9)	-0.9(10)	5.8(7)	0.7(9)	**C5**	23.5(13)	25.1(19)	25.3(13)	3.5(13)	12.5(10)	-1.5(12)
**C6**	21.4(10)	21.4(15)	20.1(10)	-1.1(10)	6.0(8)	-5.8(10)	**C6**	24.9(13)	27.4(19)	26.6(14)	0.4(13)	13.3(11)	1.8(13)
**C7**	24.7(10)	13.1(14)	17.7(9)	-1.2(9)	8.1(8)	-3.9(9)	**C7**	29.2(13)	16.4(18)	23.3(13)	-1.1(12)	13.7(11)	3.1(12)
**N8**	19.2(8)	10.7(11)	12.9(8)	2.2(8)	3.4(6)	-0.2(7)	**N8**	22.0(10)	12.5(15)	20.5(11)	-2.8(10)	8.4(9)	-1.1(10)
**C9**	21.8(10)	9.4(13)	14.1(9)	-0.9(8)	2.4(7)	0.3(9)	**C9**	26.5(13)	15.3(17)	17.9(12)	-0.6(11)	7.6(10)	-1.8(12)
**C10**	24.6(10)	13.3(14)	13.4(9)	0.4(8)	3.4(7)	0.0(9)	**C10**	30.0(13)	15.3(17)	19.0(12)	-2.2(11)	10.6(11)	-1.4(12)
**O11**	30.2(8)	19.1(11)	19.5(7)	-6.2(7)	3.8(6)	-0.4(7)	**O11**	33.5(11)	25.5(14)	24.9(10)	7.5(9)	12.1(8)	3.4(9)
**C12**	22.7(10)	15.6(14)	19.7(10)	-3.4(9)	-0.4(8)	-1.6(9)	**C12**	26.3(13)	20.0(18)	26.0(14)	4.9(13)	6.5(11)	0.0(12)
**C13**	20.7(10)	15.6(14)	18.9(9)	-3.7(9)	-2.5(8)	-3.7(9)	**C13**	20.5(12)	20.9(18)	24.3(13)	4.8(12)	3.6(10)	3.3(12)
**C14**	27.1(11)	16.4(14)	22.4(10)	-2.4(10)	-2.0(8)	-3.7(10)	**C14**	30.5(14)	25.9(19)	26.5(14)	-2.9(13)	7.2(12)	0.5(14)
**C15**	27.7(11)	18.5(15)	24.9(11)	-2.8(10)	1.8(9)	-4.1(10)	**C15**	29.9(14)	23.1(19)	26.7(13)	2.9(13)	7.8(11)	3.6(13)
**C16**	30.1(12)	20.5(16)	38.4(13)	-6.1(11)	-7.6(10)	3.4(11)	**C16**	34.0(16)	34(2)	39.9(17)	1.8(15)	19.5(14)	4.0(15)
**C17**	28.7(12)	36(2)	40.5(14)	-10.2(13)	13.1(11)	-8.4(12)	**C17**	30.9(15)	24(2)	37.1(17)	-2.3(15)	2.7(13)	-3.9(13)
**C18**	22.1(11)	32.6(19)	52.7(15)	-14.4(15)	5.0(10)	0.5(12)	**C18**	27.7(14)	31(2)	49.7(19)	7.0(17)	16.1(14)	-0.4(14)
**N19**	23.3(9)	11.6(11)	15.3(8)	-1.2(7)	5.0(6)	1.3(8)	**N19**	26.3(11)	16.1(14)	21.8(11)	1.4(10)	11.4(9)	-2.9(10)
**C20**	19.8(9)	8.7(13)	17.6(9)	0.0(8)	5.0(7)	0.7(8)	**C20**	24.5(13)	14.6(16)	24.2(13)	-0.1(12)	11.4(11)	-1.2(11)
**C21**	14.3(9)	8.9(13)	17.7(9)	-1.1(9)	2.6(7)	-0.8(8)	**C21**	16.7(11)	17.0(18)	25.1(13)	-1.7(12)	9.4(10)	-1.2(11)
**O22**	23.9(7)	10.8(10)	18.5(7)	0.3(7)	4.0(5)	0.2(6)	**O22**	26.4(10)	18.9(13)	28.8(10)	-2.9(9)	10.5(8)	-1.7(9)
**C23**	20.4(10)	19.7(15)	18.8(9)	1.8(9)	6.6(8)	-0.6(9)	**C23**	26.2(14)	24.9(19)	27.7(14)	-4.4(13)	15.0(12)	-1.9(13)
**C24**	24.6(10)	19.4(15)	21.2(10)	1.6(10)	8.4(8)	4.8(10)	**C24**	33.1(15)	24(2)	31.3(15)	-3.2(13)	18.7(12)	-6.9(13)
**C25**	27.4(11)	12.4(14)	18.8(9)	-0.9(9)	8.2(8)	4.6(10)	**C25**	33.2(14)	19.3(18)	27.3(13)	0.2(13)	17.9(11)	-5.5(13)
**N26**	19.4(8)	8.2(12)	16.0(8)	2.9(8)	4.3(6)	-0.9(7)	**N26**	24.6(11)	14.9(16)	22.2(12)	-3.3(10)	8.1(9)	-0.4(10)
**C27**	18.7(9)	9.8(13)	14.3(9)	0.0(8)	2.6(7)	-0.1(9)	**C27**	22.8(13)	29.7(19)	20.9(13)	-2.8(12)	6.8(11)	1.6(12)
**C28**	23.4(10)	10.1(13)	14.1(9)	-0.3(9)	5.1(7)	0.7(9)	**C28**	26.0(13)	16.9(17)	20.2(13)	4.0(12)	8.4(10)	1.3(12)
**O29**	25.5(7)	16.9(10)	19.7(7)	6.2(7)	3.2(6)	-0.6(7)	**O29**	29.8(10)	27.8(14)	23.8(10)	-7.2(9)	8.3(8)	-1.3(9)
**C30**	19.5(10)	13.7(13)	18.0(9)	2.5(9)	1.0(8)	0.4(9)	**C30**	30.0(14)	27(2)	26.4(14)	3.3(13)	9.5(12)	0.8(13)
**C31**	18.3(9)	13.2(13)	18.0(9)	3.4(9)	-0.4(7)	1.5(9)	**C31**	26.1(13)	26.7(19)	25.4(14)	2.9(13)	5.2(11)	4.2(13)
**C32**	25.2(10)	22.1(16)	22.4(10)	-0.5(10)	3.6(8)	1.6(10)	**C32**	31.8(15)	28(2)	34.4(16)	-0.7(14)	10.8(12)	-7.1(14)
**C33**	21.1(10)	15.4(14)	22.3(9)	1.2(9)	-0.1(8)	3.3(9)	**C33**	36.9(16)	32(2)	33.7(16)	-1.9(15)	9.2(13)	-4.4(15)
**C34**	26.3(11)	32.8(18)	32.0(12)	5.1(11)	10.9(9)	0.3(11)	**C34**	51(2)	34(2)	46.7(19)	-3.9(17)	28.4(17)	2.8(17)
**C35**	24.8(11)	13.2(15)	34.3(12)	-0.1(10)	-2.1(9)	-0.2(10)	**C35**	36.0(17)	50(3)	39.5(18)	1.7(19)	10.0(14)	-12.6(18)
**C36**	22.2(11)	23.1(16)	43.3(14)	7.6(12)	4.7(10)	-4.6(11)	**C36**	26.9(15)	65(3)	46.4(19)	13(2)	21.3(14)	1.2(17)

Table S3: Bond Lengths in Å for **c(Pro-Phe-Pro-Phe)** and **c(Pro-Phe-Pro-*D*-Phe)**, respectively.

c(Pro-Phe-Pro-Phe)			c(Pro-Phe-Pro-*D*-Phe)		
**Atom**	**Atom**	**Length [Å]**	**Atom**	**Atom**	**Length [Å]**
**N1**	**C2**	1.469(3)	**N1**	**C2**	1.477(4)
**N1**	**C7**	1.480(3)	**N1**	**C7**	1.483(3)
**N1**	**C28**	1.364(3)	**N1**	**C28**	1.357(3)
**C2**	**C3**	1.533(3)	**C2**	**C3**	1.535(3)
**C2**	**C5**	1.555(3)	**C2**	**C5**	1.554(3)
**C3**	**O4**	1.228(3)	**C3**	**O4**	1.229(4)
**C3**	**N8**	1.341(3)	**C3**	**N8**	1.338(4)
**C5**	**C6**	1.527(4)	**C5**	**C6**	1.523(5)
**C6**	**C7**	1.517(3)	**C6**	**C7**	1.512(4)
**N8**	**C9**	1.465(2)	**N8**	**C9**	1.469(3)
**C9**	**C10**	1.541(3)	**C9**	**C10**	1.539(4)
**C9**	**C12**	1.540(3)	**C9**	**C12**	1.533(4)
**C10**	**O11**	1.229(3)	**C10**	**O11**	1.226(3)
**C10**	**N19**	1.352(3)	**C10**	**N19**	1.348(4)
**C12**	**C13**	1.509(3)	**C12**	**C13**	1.508(4)
**C13**	**C14**	1.391(4)	**C13**	**C14**	1.395(4)
**C13**	**C15**	1.393(3)	**C13**	**C15**	1.384(5)
**C14**	**C16**	1.391(4)	**C14**	**C16**	1.387(5)
**C15**	**C17**	1.391(4)	**C15**	**C17**	1.389(5)
**C16**	**C18**	1.387(4)	**C16**	**C18**	1.380(5)
**C17**	**C18**	1.378(5)	**C17**	**C18**	1.380(5)
**N19**	**C20**	1.458(3)	**N19**	**C20**	1.452(4)
**N19**	**C25**	1.474(3)	**N19**	**C25**	1.474(3)
**C20**	**C23**	1.549(3)	**C20**	**C23**	1.549(4)
**C21**	**O22**	1.225(3)	**C21**	**O22**	1.225(4)
**C21**	**N26**	1.349(3)	**C21**	**N26**	1.346(4)
**C23**	**C24**	1.533(4)	**C23**	**C24**	1.533(5)
**C24**	**C25**	1.524(3)	**C24**	**C25**	1.523(4)
**N26**	**C27**	1.462(2)	**N26**	**C27**	1.452(4)
**C27**	**C28**	1.538(3)	**C27**	**C28**	1.536(4)
**C27**	**C30**	1.536(3)	**C27**	**C30**	1.516(4)
**C28**	**O29**	1.229(3)	**C28**	**O29**	1.225(4)
**C30**	**C31**	1.508(3)	**C30**	**C31**	1.523(4)
**C31**	**C32**	1.399(3)	**C31**	**C32**	1.378(5)
**C31**	**C33**	1.390(3)	**C31**	**C33**	1.382(5)
**C32**	**C34**	1.389(4)	**C32**	**C34**	1.396(4)
**C33**	**C35**	1.387(3)	**C33**	**C35**	1.375(5)
**C34**	**C36**	1.383(4)	**C34**	**C36**	1.392(6)
**C35**	**C36**	1.389(4)	**C35**	**C36**	1.368(6)

Table S4: Bond Angles in for **c(Pro-Phe-Pro-Phe)** and **c(Pro-Phe-Pro-*D*-Phe)**, respectively.

c(Pro-Phe-Pro-Phe)				c(Pro-Phe-Pro-*D*-Phe)			
**Atom**	**Atom**	**Atom**	**Angle [°]**	**Atom**	**Atom**	**Atom**	**Angle [°]**
C28	N1	C2	125.52(18)	C28	N1	C2	126.5(2)
C28	N1	C7	116.57(17)	C28	N1	C7	115.8(2)
C2	N1	C7	111.49(17)	C2	N1	C7	110.8(2)
C3	N8	C9	121.32(19)	C3	N8	C9	121.4(3)
C21	N26	C27	121.1(2)	C21	N26	C27	119.3(3)
C20	N19	C25	112.39(17)	C20	N19	C25	112.7(2)
C10	N19	C20	127.11(18)	C10	N19	C20	128.1(2)
C10	N19	C25	118.23(18)	C10	N19	C25	119.0(2)
O29	C28	N1	120.7(2)	O29	C28	N1	121.1(2)
O29	C28	C27	120.63(18)	O29	C28	C27	116.3(2)

c(Pro-Phe-Pro-Phe)				c(Pro-Phe-Pro-*D*-Phe)			
**Atom**	**Atom**	**Atom**	**Angle [˚]**	**Atom**	**Atom**	**Atom**	**Angle [˚]**
N1	C28	C27	118.61(18)	N1	C28	C27	122.4(2)
O22	C21	N26	123.43(18)	O22	C21	N26	122.6(3)
O22	C21	C20	123.03(19)	O22	C21	C20	122.9(3)
N26	C21	C20	113.5(2)	N26	C21	C20	114.5(3)
O4	C3	N8	123.84(18)	O4	C3	N8	123.7(2)
O4	C3	C2	117.2(2)	O4	C3	C2	116.3(3)
N8	C3	C2	118.9(2)	N8	C3	C2	119.9(3)
C32	C31	C30	120.9(2)	C32	C31	C30	121.0(3)
C33	C31	C30	120.70(19)	C33	C31	C30	120.2(3)
C33	C31	C32	118.3(2)	C33	C31	C32	118.8(3)
N19	C20	C21	111.75(18)	N19	C20	C21	111.6(2)
N19	C20	C23	102.61(17)	N19	C20	C23	103.0(2)
C21	C20	C23	109.03(16)	C21	C20	C23	108.5(2)
N1	C2	C3	117.84(18)	N1	C2	C3	119.6(2)
N1	C2	C5	103.23(17)	N1	C2	C5	103.0(2)
C3	C2	C5	108.26(16)	C3	C2	C5	107.1(2)
N1	C7	C6	102.66(17)	N1	C7	C6	102.9(2)
O11	C10	N19	120.66(19)	O11	C10	N19	120.6(3)
O11	C10	C9	120.36(19)	O11	C10	C9	120.2(2)
N19	C10	C9	118.96(19)	N19	C10	C9	119.1(3)
C31	C30	C27	114.24(19)	C31	C30	C27	110.2(2)
N26	C27	C28	112.23(16)	N26	C27	C28	115.1(2)
N26	C27	C30	111.88(16)	N26	C27	C30	111.2(3)
C30	C27	C28	109.24(18)	C30	C27	C28	114.2(2)
C15	C13	C12	120.4(2)	C15	C13	C12	120.9(3)
C14	C13	C12	121.1(2)	C14	C13	C12	120.8(3)
C14	C13	C15	118.3(2)	C14	C13	C15	118.2(3)
C34	C32	C31	120.6(2)	C34	C32	C31	120.6(3)
N19	C25	C24	102.99(18)	N19	C25	C24	103.7(2)
C6	C5	C2	103.04(19)	C6	C5	C2	103.6(2)
C35	C33	C31	121.2(2)	C35	C33	C31	120.7(3)
N8	C9	C10	113.58(17)	N8	C9	C10	113.6(2)
N8	C9	C12	111.22(16)	N8	C9	C12	110.8(2)
C12	C9	C10	107.87(18)	C12	C9	C10	108.6(2)
C13	C12	C9	115.20(19)	C13	C12	C9	115.2(2)
C7	C6	C5	102.46(19)	C7	C6	C5	102.1(2)
C24	C23	C20	102.68(19)	C24	C23	C20	103.0(2)
C17	C15	C13	120.7(3)	C17	C15	C13	121.2(3)
C16	C14	C13	120.9(2)	C16	C14	C13	120.7(3)
C25	C24	C23	101.98(19)	C25	C24	C23	103.2(2)
C36	C34	C32	120.2(2)	C36	C34	C32	119.6(3)
C34	C36	C35	119.8(2)	C34	C36	C35	119.2(3)
C33	C35	C36	119.9(2)	C33	C35	C36	121.0(3)
C18	C16	C14	119.9(3)	C18	C16	C14	120.3(3)
C17	C18	C16	119.7(2)	C17	C18	C16	119.6(3)
C18	C17	C15	120.3(3)	C18	C17	C15	120.0(3)

Table S5: Torsion Angles in for **c(Pro-Phe-Pro-Phe)** and **c(Pro-Phe-Pro-*D*-Phe)**, respectively.

c(Pro-Phe-Pro-Phe)					c(Pro-Phe-Pro-*D*-Phe)				
**A**	**B**	**C**	**D**	**Angle [˚]**	**A**	**B**	**C**	**D**	**Angle [˚]**
O22	C21	C20	N19	-26.5(3)	O22	C21	C20	N19	-26.2(3)
O22	C21	C20	C23	86.2(2)	O22	C21	C20	C23	86.6(3)
O29	C28	C27	N26	-137.0(2)	O29	C28	C27	N26	-155.3(3)
O29	C28	C27	C30	-12.3(3)	O29	C28	C27	C30	74.1(4)
O4	C3	C2	N1	157.54(19)	O4	C3	C2	N1	158.2(2)
O4	C3	C2	C5	-85.9(2)	O4	C3	C2	C5	-85.3(3)

c(Pro-Phe-Pro-Phe)					c(Pro-Phe-Pro-*D*-Phe)				
**A**	**B**	**C**	**D**	**Angle [˚]**	**A**	**B**	**C**	**D**	**Angle [˚]**
O11	C10	C9	N8	-136.6(2)	O11	C10	C9	N8	-133.3(3)
O11	C10	C9	C12	-12.8(3)	O11	C10	C9	C12	-9.5(4)
N1	C28	C27	N26	44.8(3)	N1	C28	C27	N26	19.6(4)
N1	C28	C27	C30	169.53(19)	N1	C28	C27	C30	-110.9(3)
N1	C2	C5	C6	27.40(19)	N1	C2	C5	C6	26.5(3)
N1	C7	C6	C5	37.8(2)	N1	C7	C6	C5	38.9(3)
N8	C3	C2	N1	-25.2(3)	N8	C3	C2	N1	-25.5(3)
N8	C3	C2	C5	91.4(2)	N8	C3	C2	C5	91.0(3)
N8	C9	C12	C13	-58.4(3)	N8	C9	C12	C13	-62.8(3)
N26	C21	C20	N19	154.66(17)	N26	C21	C20	N19	156.9(2)
N26	C21	C20	C23	-92.6(2)	N26	C21	C20	C23	-90.3(3)
N19	C20	C23	C24	31.9(2)	N19	C20	C23	C24	31.4(3)
N19	C10	C9	N8	44.9(3)	N19	C10	C9	N8	50.7(4)
N19	C10	C9	C12	168.70(19)	N19	C10	C9	C12	174.5(2)
N19	C25	C24	C23	35.0(2)	N19	C25	C24	C23	30.2(3)
C28	N1	C2	C3	-93.9(2)	C28	N1	C2	C3	-94.1(3)
C28	N1	C2	C5	146.8(2)	C28	N1	C2	C5	147.3(3)
C28	N1	C7	C6	-174.90(19)	C28	N1	C7	C6	-176.2(3)
C21	N26	C27	C28	53.4(2)	C21	N26	C27	C28	74.1(3)
C21	N26	C27	C30	-69.8(2)	C21	N26	C27	C30	-153.9(2)
C21	C20	C23	C24	-86.7(2)	C21	C20	C23	C24	-87.0(3)
C3	N8	C9	C10	-130.5(2)	C3	N8	C9	C10	-129.4(3)
C3	N8	C9	C12	107.6(2)	C3	N8	C9	C12	108.0(3)
C3	C2	C5	C6	-98.2(2)	C3	C2	C5	C6	-100.5(3)
C31	C30	C27	N26	-57.9(3)	C31	C30	C27	N26	69.8(3)
C31	C30	C27	C28	177.20(17)	C31	C30	C27	C28	-157.7(3)
C31	C32	C34	C36	0.0(4)	C31	C32	C34	C36	-2.0(5)
C31	C33	C35	C36	-0.6(3)	C31	C33	C35	C36	-0.9(6)
C20	N19	C10	O11	-165.9(2)	C20	N19	C10	O11	-175.9(3)
C20	N19	C10	C9	12.5(3)	C20	N19	C10	C9	0.1(4)
C20	N19	C25	C24	-15.6(2)	C20	N19	C25	C24	-10.7(3)
C20	C23	C24	C25	-41.65(19)	C20	C23	C24	C25	-38.3(3)
C2	N1	C28	O29	-160.9(2)	C2	N1	C28	O29	-153.2(3)
C2	N1	C28	C27	17.3(3)	C2	N1	C28	C27	32.1(4)
C2	N1	C7	C6	-21.4(2)	C2	N1	C7	C6	-23.2(3)
C2	C5	C6	C7	-40.62(19)	C2	C5	C6	C7	-40.7(3)
C7	N1	C28	O29	-11.6(3)	C7	N1	C28	O29	-5.1(4)
C7	N1	C28	C27	166.60(18)	C7	N1	C28	C27	-179.8(3)
C7	N1	C2	C3	115.41(19)	C7	N1	C2	C3	116.5(3)
C7	N1	C2	C5	-3.8(2)	C7	N1	C2	C5	-2.1(3)
C10	N19	C20	C21	-91.2(2)	C10	N19	C20	C21	-81.5(3)
C10	N19	C20	C23	152.1(2)	C10	N19	C20	C23	162.3(3)
C10	N19	C25	C24	-179.8(2)	C10	N19	C25	C24	173.4(3)
C10	C9	C12	C13	176.43(18)	C10	C9	C12	C13	171.8(2)
C30	C31	C32	C34	176.9(2)	C30	C31	C32	C34	-178.3(3)
C30	C31	C33	C35	-176.6(2)	C30	C31	C33	C35	179.7(3)
C27	N26	C21	O22	-2.4(3)	C27	N26	C21	O22	-5.4(4)
C27	N26	C21	C20	176.46(16)	C27	N26	C21	C20	171.5(2)
C13	C15	C17	C18	0.1(4)	C13	C15	C17	C18	-0.4(5)
C13	C14	C16	C18	-0.5(4)	C13	C14	C16	C18	-0.8(5)
C32	C31	C30	C27	101.8(2)	C32	C31	C30	C27	-101.7(4)
C32	C31	C33	C35	0.8(3)	C32	C31	C33	C35	-0.5(5)
C32	C34	C36	C35	0.2(4)	C32	C34	C36	C35	0.6(6)
C25	N19	C20	C21	106.38(19)	C25	N19	C20	C21	103.1(3)
C25	N19	C20	C23	-10.3(2)	C25	N19	C20	C23	-13.1(3)
C25	N19	C10	O11	-4.4(3)	C25	N19	C10	O11	-0.7(4)

c(Pro-Phe-Pro-Phe)					c(Pro-Phe-Pro-D-Phe)				
A	B	C	D	Angle [˚]	A	B	C	D	Angle [˚]
C25	N19	C10	C9	174.03(19)	C25	N19	C10	C9	175.3(2)
C33	C31	C30	C27	-80.8(2)	C33	C31	C30	C27	78.1(4)
C33	C31	C32	C34	-0.5(3)	C33	C31	C32	C34	1.9(5)
C9	N8	C3	O4	5.3(3)	C9	N8	C3	O4	5.5(4)
C9	N8	C3	C2	-171.75(16)	C9	N8	C3	C2	-170.6(2)
C12	C13	C15	C17	174.8(2)	C12	C13	C15	C17	-176.1(3)
C12	C13	C14	C16	-174.6(2)	C12	C13	C14	C16	176.6(3)
C15	C13	C12	C9	108.4(2)	C15	C13	C12	C9	-77.2(3)
C15	C13	C14	C16	1.2(3)	C15	C13	C14	C16	0.2(4)
C14	C13	C12	C9	-75.9(3)	C14	C13	C12	C9	-77.2(3)
C14	C13	C15	C17	-1.1(3)	C14	C13	C15	C17	0.4(4)
C14	C16	C18	C17	-0.5(4)	C14	C16	C18	C17	0.8(5)
C34	C36	C35	C33	0.1(4)	C34	C36	C35	C33	0.8(6)
C16	C18	C17	C15	0.7(4)	C16	C18	C17	C15	-0.2(5)

Table S6: Hydrogen Fractional Atomic Coordinates (×10^4) and Equivalent Isotropic Displacement Parameters ($Å^2$×10^3) for **c(Pro-Phe-Pro-Phe)** and **c(Pro-Phe-Pro-*D*-Phe)**, respectively. U_{eq} is defined as 1/3 of the trace of the orthogonalized U_{ij}.

c(Pro-Phe-Pro-Phe)					c(Pro-Phe-Pro-D-Phe)				
Atom	x	y	z	U_{eq}	Atom	x	y	z	U_{eq}
H2	5692.11	2614.66	1532.34	17	H2	4760.1	2166.1	3494.81	23
H5A	7709.5	3580.39	2086.58	23	H5A	2630.25	3026.26	3019.45	28
H5B	7272.24	3071.87	1172.45	23	H5B	3268.04	2414.52	3897.58	28
H6A	7563.38	7967.82	1926.89	25	H6A	2761.57	7427.01	3226.55	30
H6B	7998.06	7118.97	1166.89	25	H6B	2504.78	6400.72	3977.73	30
H7A	6265.5	9593.29	944.53	22	H7A	4237.41	9020.71	4178.57	26
H7B	6327.2	7193.67	385.56	22	H7B	4329.71	6475.84	4687.2	26
H8	6030(20)	7950(60)	2741(16)	17(7)	H8	4060(20)	7500(70)	2304(17)	18(8)
H9	6252.25	4988.43	4047.95	18	H9	3512.84	4493.15	1034.03	24
H12A	7402.38	9606.28	3967.26	24	H12A	2355.88	9018.44	1209.47	29
H12B	7464.2	7963.92	4738.87	24	H12B	2129.78	7521.83	423.93	29
H14	8242.72	3428.08	4714.96	28	H14	1353.25	7891.37	2047.32	34
H15	8646.21	8876.9	3182.6	29	H15	1400.64	2943.51	381.67	32
H16	9757.48	1365.98	4505.07	38	H16	-2.05	5455.53	2315.16	41
H17	10154.2	6799.21	2969.7	41	H17	23.78	551.11	637.53	39
H18	10721.93	3073.49	3636.02	43	H18	-685.45	1811.25	1602.65	42
H20	4343.01	4344.62	3445.58	18	H20	5571.48	3897.55	1540.49	24
H23A	2311.8	5171.33	3005.93	23	H23A	7654.33	4986.54	1865.54	30
H23B	2797.33	4712.52	3916.42	23	H23B	6981.75	4218.82	1004.99	30
H24A	2406.8	9594.25	3152.03	25	H24A	7468.47	9341.94	1599.13	33
H24B	2003.98	8709.88	3922.24	25	H24B	7637.37	8244.11	827.61	33
H25A	3727.8	11291.8	4100.61	23	H25A	5883.44	10821.82	760.27	30
H25B	3676.8	8968.93	4689.36	23	H25B	5815.2	8483.33	179.6	30
H26	3878(18)	3010(60)	2164(14)	11(6)	H26	6370(20)	3050(70)	2831(16)	10(8)
H27	3860.84	3010.73	932.56	17	H27	7362.72	6545.28	3799.64	29
H30A	2505.88	7313.45	940.04	21	H30A	7058.43	1330.2	4076.05	33
H30B	2579.98	5690.37	184.78	21	H30B	7628.1	3251.14	4757.45	33
H32	1319.76	6024.28	1756.1	28	H32	8109.11	-307.69	3300.45	38
H33	1914.24	1166.93	113.67	24	H33	9327.77	5608.73	4693.44	41
H34	-73.54	3451.2	1945.36	36	H34	9796.12	-801.18	3073.87	49
H35	513.78	-1381.6	295.5	30	H35	10969.89	5235.18	4431.94	50
H36	-481.87	-247.94	1217.02	36	H36	11232.45	2036.72	3638.56	53

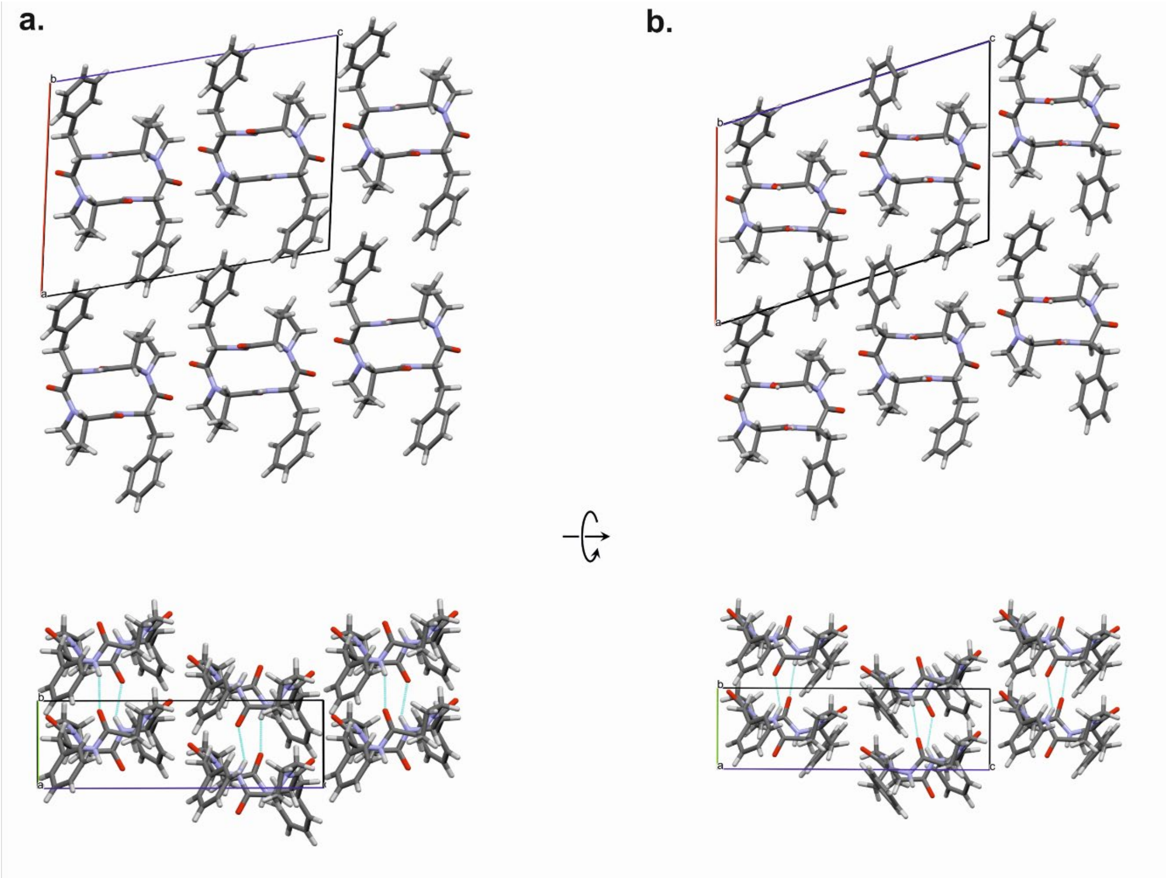

Figure S5. A crystal packing diagram of (*a*) c(Pro-Phe-Pro-Phe) (sample A) and (*b*) c(Pro-Phe-Pro-D-Phe) (sample B). The views along the *b*-axis are shown above, while the views along the *a*-axis are shown below.

3. Theoretical Calculations and ECD Spectroscopy

Table S7. Relative conformers energies with population at 298 K for sample **A** after DFT calculations at the B3LYP/6-311+G(d,p)/PCM(H_2O) level of theory.

Structure ID	Pop. [%]	ΔE [kcal/mol]
3	39.68	0
1	21.46%	0.36
2	21.31%	0.37
5	8.21%	0.93
9	3.63%	1.41
X-Ray	3.14%	1.50
4	1.41%	1.98
7	0.52%	2.57
10	0.31%	2.87
8	0.28%	2.94
6	0.05%	3.99

Table S8. Relative conformers energies with population at 298 K for sample **B** after DFT calculations at the B3LYP/6 311+G(d,p)/PCM(H_2O) level of theory.

Structure ID	Pop. [%]	ΔE [kcal/mol]
X-Ray	51.64%	0
4	40.58%	0.14
1	5.14%	1.36
2	2.25%	1.86
3	0.32%	3.02

7	0.05%	4.12
6	0.03%	4.51
5	0.00%	6.76

4. Molecular dynamics on the base of 2D NMR spectroscopy

Table S9. cPFPF sample heteriochiral B.

	Atoms												
Amino acids	C_A	C_B	C_C	C_D	H_A	H_{B1}	H_{B2}	H_{C1}	H_{C2}	H_{D1}	H_{D2}	N	NH
xPro	58.24	31.62	21.21	47.97	4.200	2.093	1.782	1.778	1.712	3.456	3.413	-	-
yPro	61.04	32.26	21.08	48.46	4.175	1.763	1.030	1.395	1.261	3.426	3.150	-	-
2Phe	59.04	35.30	-	-	4.284	3.020	2.840	-	-	-	-	116.4	8.487
4Phe	51.52	37.31	-	-	5.098	3.189	2.831	-	-	-	-	117.3	8.045

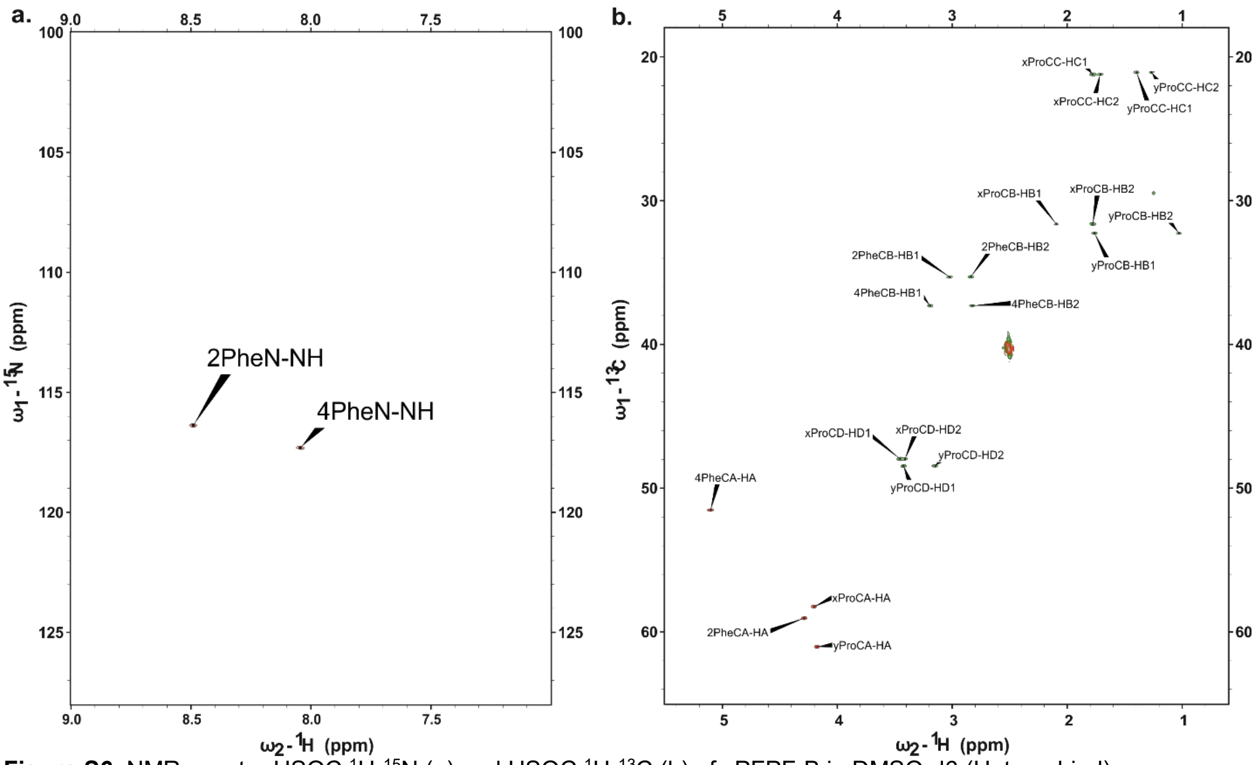

Figure S6. NMR spectra HSQC ^{1}H-^{15}N (a) and HSQC ^{1}H-^{13}C (b) of cPFPF-B in DMSO-d6 (Heterochiral).

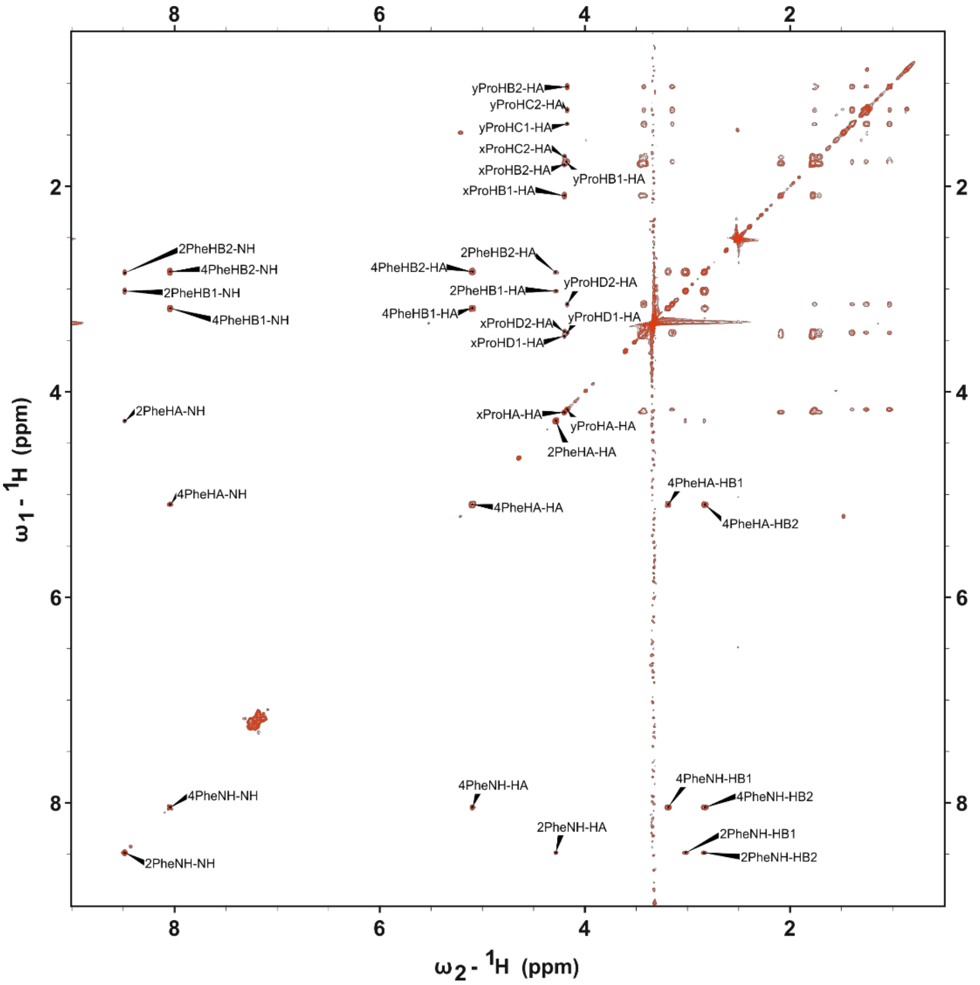

Figure S7. NMR TOCSY ^1H-^1H spectrum of cPFPF-B in DMSO-d6 (Heterochiral).

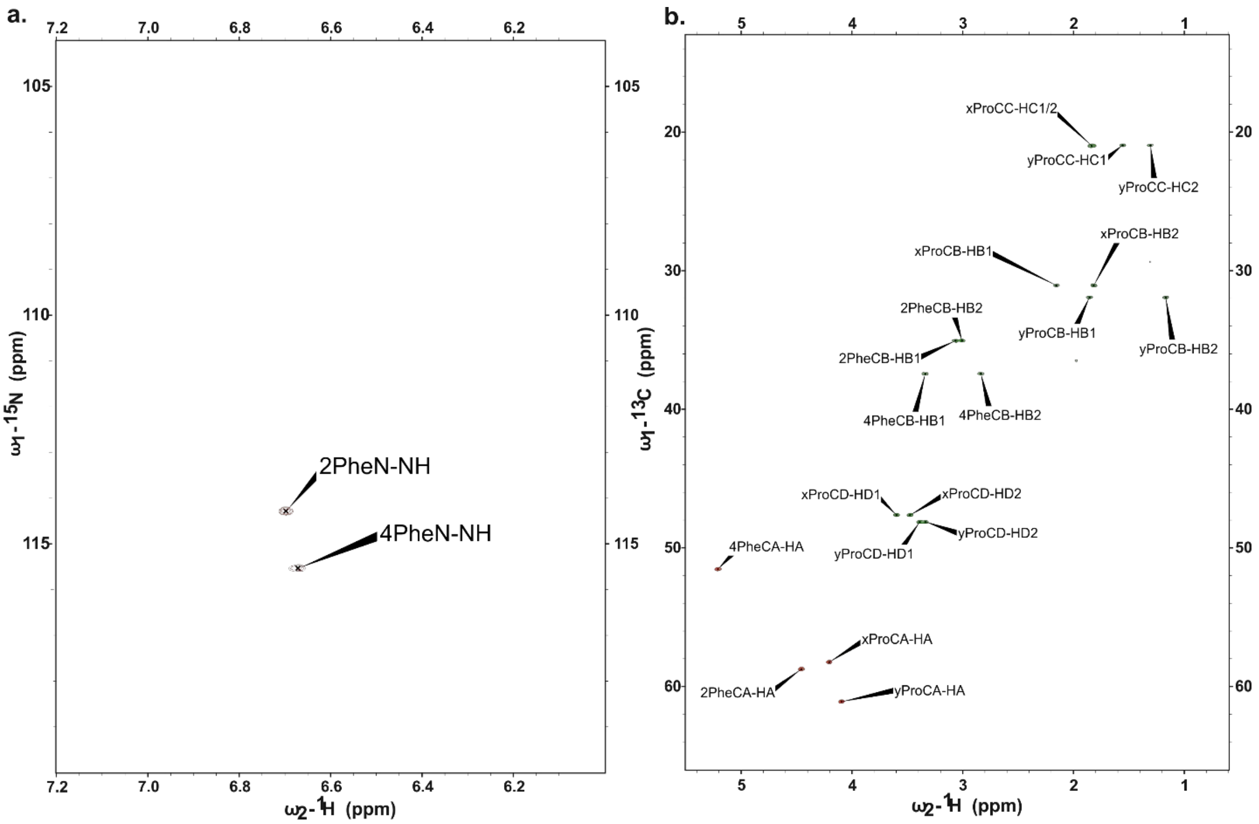

Figure S8. NMR spectra HSQC ^1H-^{15}N (a) and HSQC ^1H-^{13}C (b) of cPFPF-B in CD_3CN (Heterochiral).

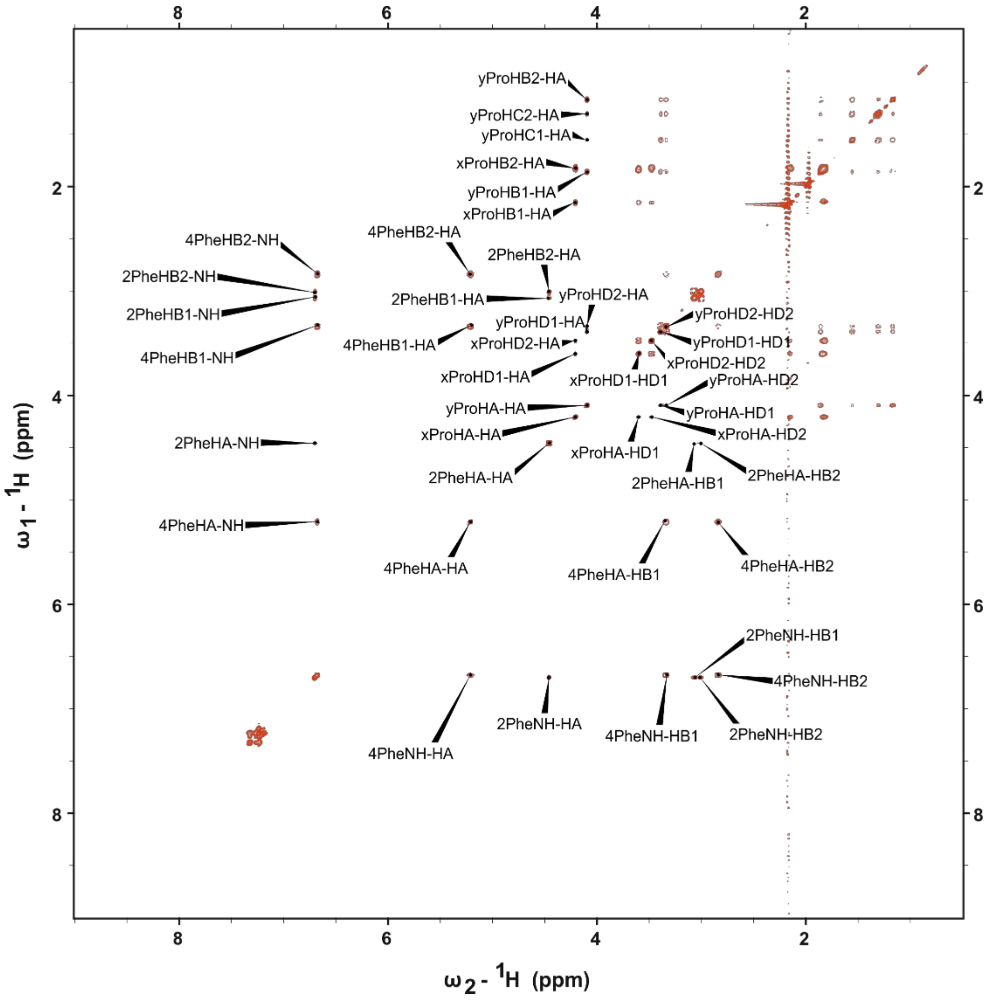

Figure S9. NMR TOCSY ^1H-^1H spectrum of cPFPF-B, CD_3CN (Heterochiral).

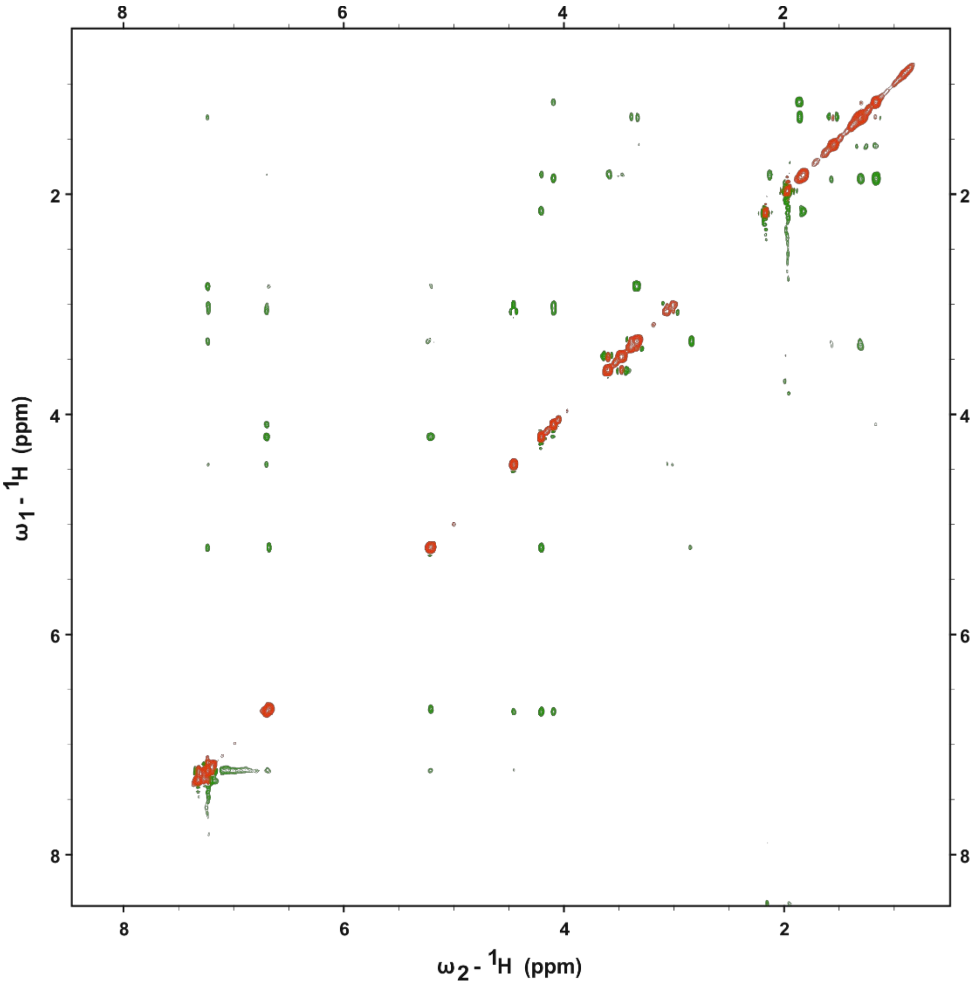

Figure S10. NMR NOESY ^1H-^1H spectrum of cPFPF-B, CD_3CN (Heterochiral).

Table S10. NMR chemical shifts of cPFPF sample homochiral A.

	Atoms													
Amino acids	C_A	C_B	C_C	C_D	H_A	H_{B1}	$H_{B1/2}$	H_{B2}	H_{C1}	H_{C2}	H_{D1}	H_{D2}	N	NH
U-xPro	62.37	31.41	21.61	47.70	4.056	1.905	-	1.239	1.676	1.541	3.503	3.420	-	-
U-yPro	58.40	30.72	21.03	47.45	3.938	1.974	-	1.658	1.953	1.885	3.603	3.427	-	-
S-1Pro	61.44	30.91	21.30	47.11	4.146	1.891	-	1.715	1.728	1.362	3.509	3.312	-	-
S-Phe	51.75	37.10	-	-	4.999	3.192	-	2.818	-	-	-	-	118.0	7.024
U-Phe	55.68	35.15	-	-	3.883	-	3.175	-	-	-	-	-	109.6	6.708
U-Phe	51.73	37.64	-	-	5.076	3.301	-	2.820	-	-	-	-	114.7	6.628

prefix S means form of peptide A with 2-fold symmetry and
prefix U means unsymmetrical form of A

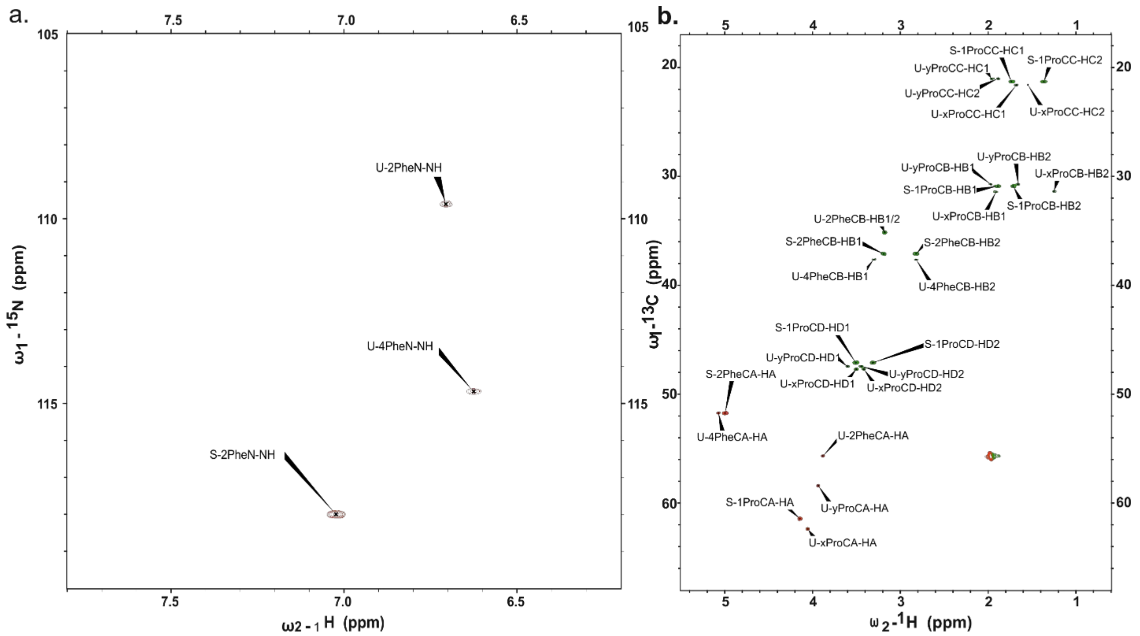

Figure S11. NMR HSQC ^1H-^{15}N (a) and HSQC ^1H-^{13}C (b) spectra of cPFPF-A, CD_3CN (Homochiral). Prefix S means form of peptide A with 2-fold symmetry and prefix U means unsymmetrical form of A.

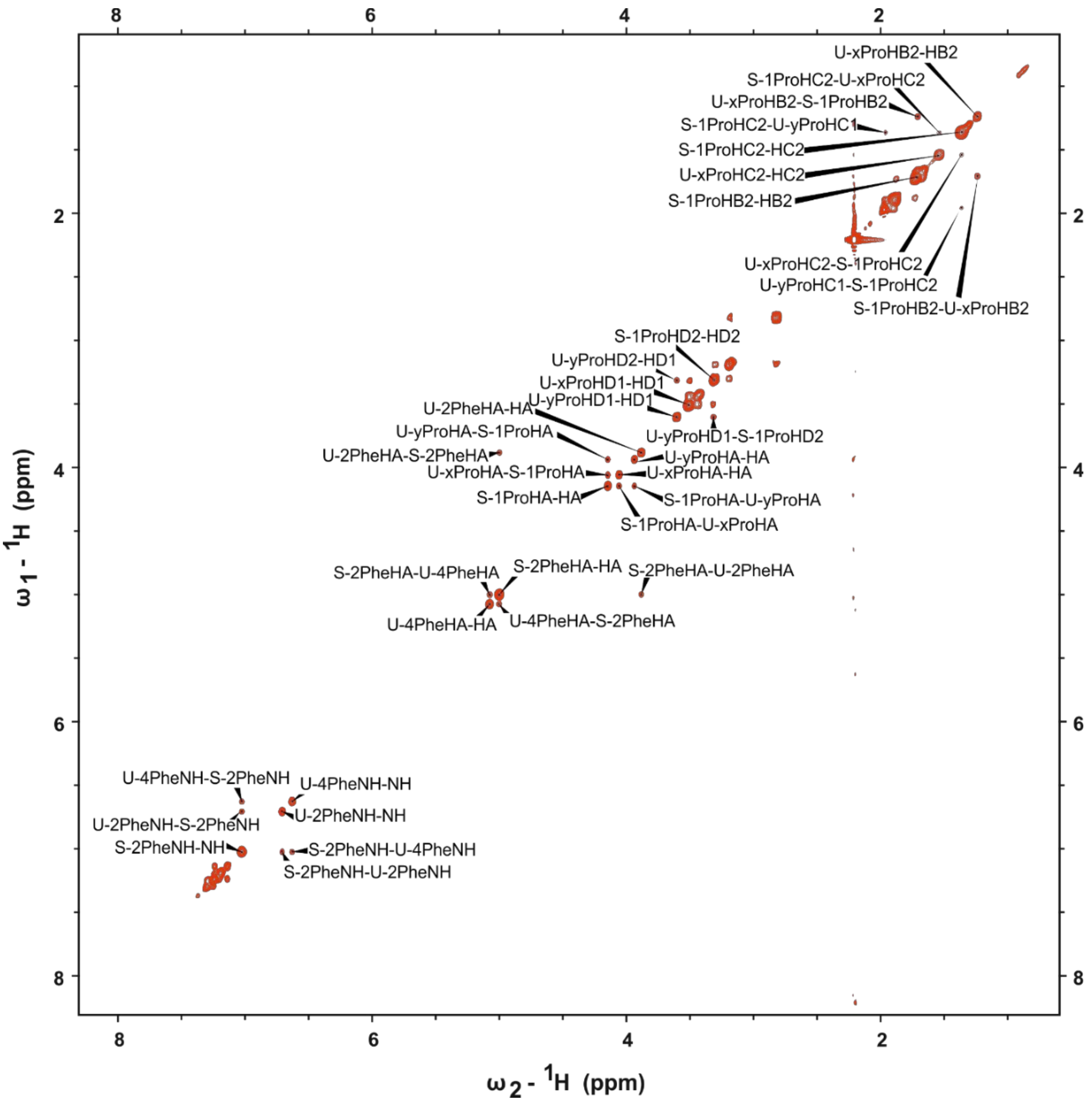

Figure S12. NMR EXSY-NOESY ^1H-^1H with mixing 50 ms spectrum of cPFPF-A in CD_3CN (Homoochiral). Showed only EXSY signals.

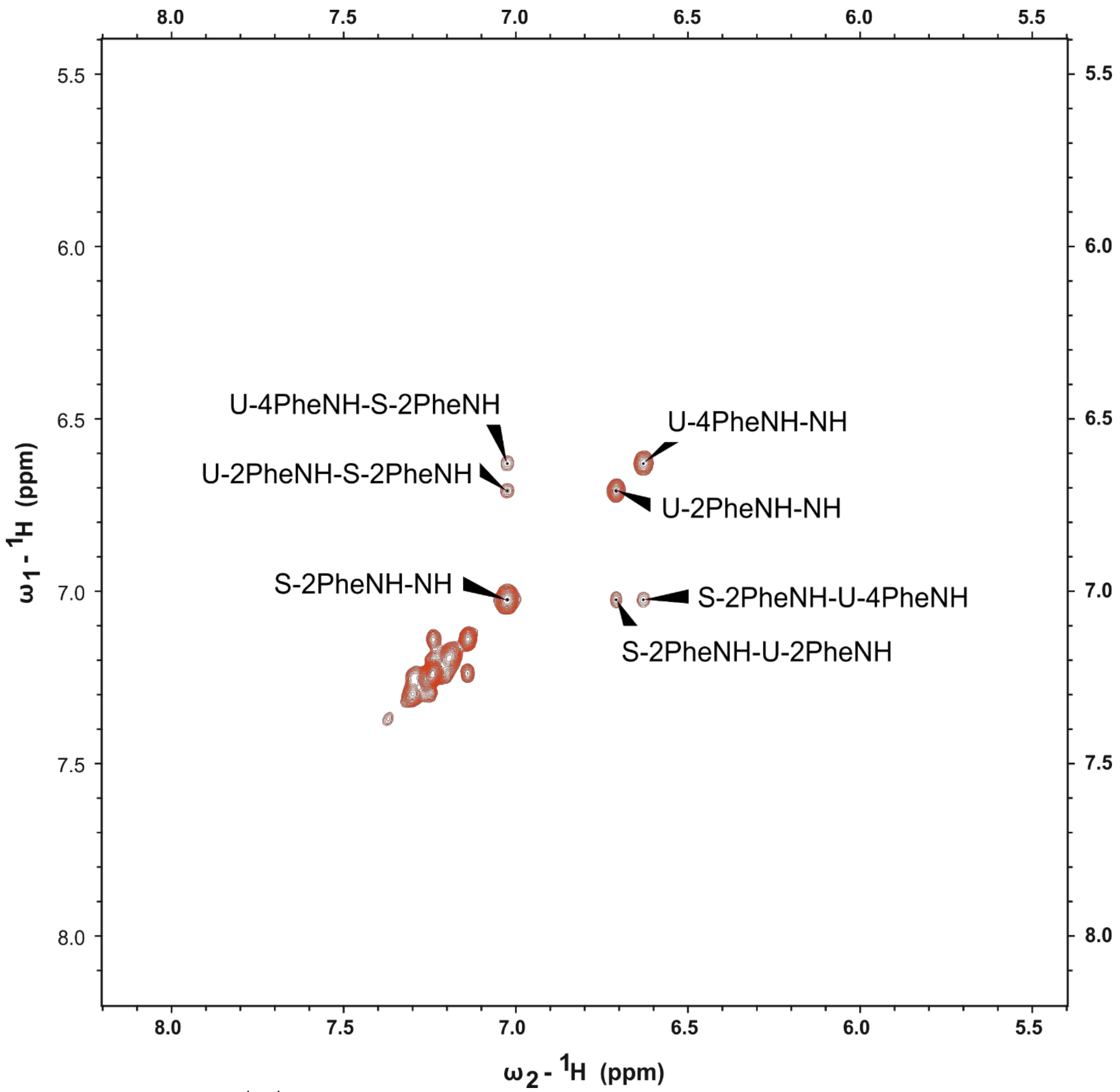

Figure S13. NMR EXSY ^{1}H-^{1}H with mixing 50 ms spectrum of cPFPF-A in CD_3CN ACN (Homoochiral).

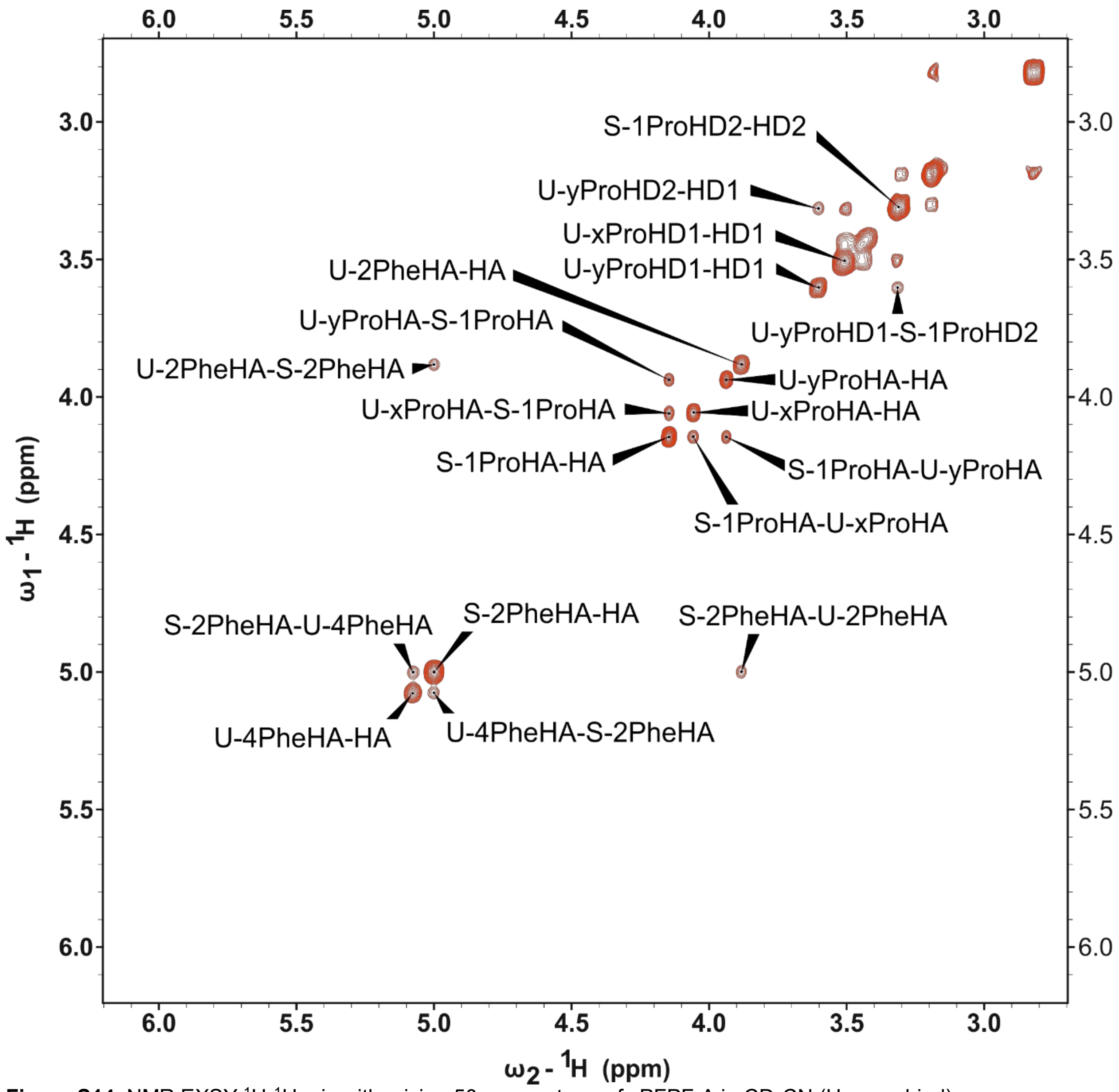

Figure S14. NMR EXSY ^1H-^1H mix with mixing 50ms spectrum of cPFPF-A in CD_3CN (Homoochiral).

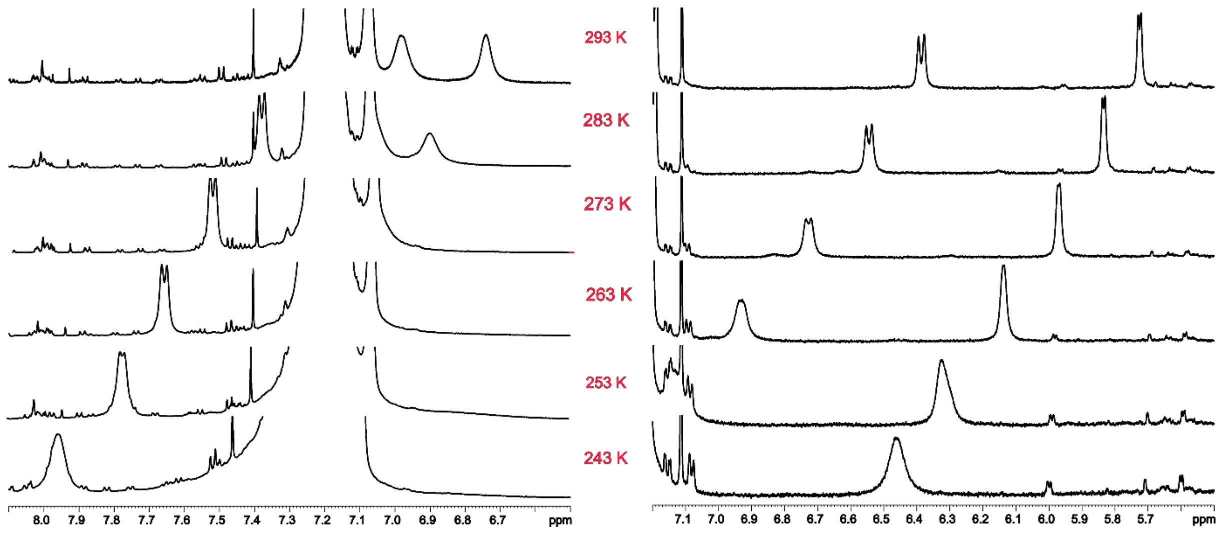

Figure S15. 600 MHz ^1H NMR spectra of N-H region of sample A (left) and sample B (right) dissolved in chloroform-d. The spectra were recorded between 243 and 293 K with 10 K increment.

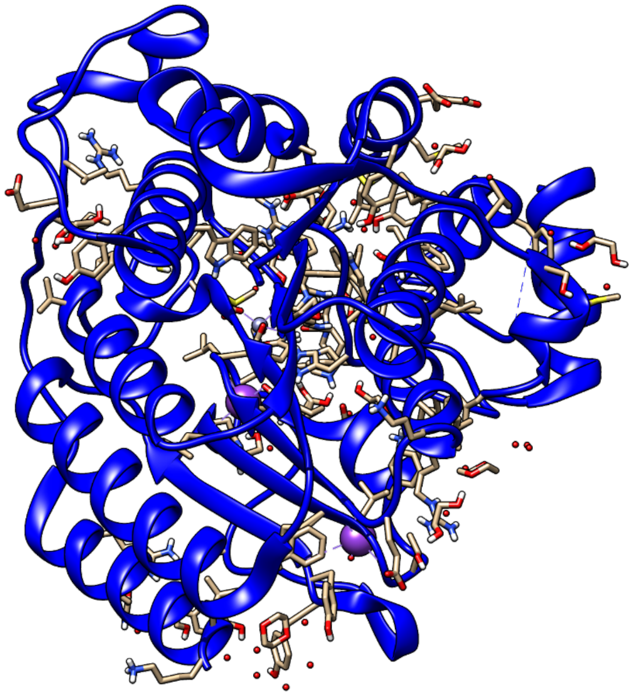

Figure S16. Crystal structure of histone deacetylase 8 in complex with trapoxin A.
